# Correlation Between Circulating Tumor Cell DNA Genomic Alterations and Mesenchymal CTCs or CTC-Associated White Blood Cell Clusters in Hepatocellular Carcinoma

**DOI:** 10.3389/fonc.2021.686365

**Published:** 2021-06-11

**Authors:** Chunming Wang, Qiong Luo, Wenbin Huang, Cheng Zhang, Hangyu Liao, Kunling Chen, MingXin Pan

**Affiliations:** ^1^ General Surgery Center, Department of Hepatobiliary Surgery II, Guangdong Provincial Research Center for Artificial Organ and Tissue Engineering, Guangzhou Clinical Research and Transformation Center for Artificial Liver, Institute of Regenerative Medicine, Zhujiang Hospital, Southern Medical University, Guangzhou, China; ^2^ Department of General Surgery, Affiliated Hengyang Hospital, Southern Medical University (Hengyang Central Hospital), Hengyang, China

**Keywords:** CTC-associated WBC (CTC-WBC) cluster, hepatocellular carcinoma, mesenchymal CTC, liquid biopsy, next-generation sequencing (NGS)

## Abstract

**Purpose:**

Liquid biopsy is attracting attention as a method of real-time monitoring of patients with tumors. It can be used to understand the temporal and spatial heterogeneity of tumors and has good clinical application prospects. We explored a new type of circulating tumor cell (CTC) enrichment technology combined with next-generation sequencing (NGS) to analyze the correlation between genomic alterations in circulating tumor cells of hepatocellular carcinoma and the counts of mesenchymal CTCs and CTC-associated white blood cell (CTC-WBC) clusters.

**Methods:**

We collected peripheral blood samples from 29 patients with hepatocellular carcinoma from January 2016 to December 2019. We then used the CanPatrol™ system to capture and analyze mesenchymal CTCs and CTC-WBC clusters for all the patients. A customized Illumina panel was used for DNA sequencing and the Mann–Whitney U test was used to test the correlation between mesenchymal CTCs, CTC-WBC cluster counts, and specific genomic changes.

**Results:**

At least one somatic hotspot mutation was detected in each of the 29 sequenced patients. A total of 42 somatic hot spot mutations were detected in tumor tissue DNA, and 39 mutations were detected in CTC-DNA, all of which included common changes in PTEN, MET, EGFR, RET, and FGFR3. The number of mesenchymal CTCs was positively correlated with the somatic genomic alterations in the PTEN and MET genes (PTEN, P = 0.021; MET, P  = 0.008, Mann–Whitney U test) and negatively correlated with the somatic genomic alterations in the EGFR gene (P = 0.006, Mann–Whitney U test). The number of CTC-WBC clusters was positively correlated with the somatic genomic alterations in RET genes (P  = 0.01, Mann–Whitney U test) and negatively correlated with the somatic genomic alterations in FGFR3 (P = 0.039, Mann–Whitney U test).

**Conclusions:**

We report a novel method of a CTC enrichment platform combined with NGS technology to analyze genetic variation, which further demonstrates the potential clinical application of this method for spatiotemporal heterogeneity monitoring of hepatocellular carcinoma. We found that the number of peripheral blood mesenchymal CTCs and CTC-WBC clusters in patients with hepatocellular carcinoma was related to a specific genome profile.

## Introduction

Most tumor-related deaths are caused by metastasis, which is a process by which tumor cells escape from the original tumor site and enter the blood circulation to spread to other organs. Tumor cells found in circulation are called circulating tumor cells (CTCs). CTCs can be divided into different subtypes, including epithelial CTCs, mesenchymal CTCs, and mixed CTCs (epithelial/mesenchymal) (1, 2). However, many CTC detection technologies, including Cell-Search system, the only clinical application approved by the U.S. Food and Drug Administration (FDA), are based only on the detection and separation of CTCs based on epithelial markers, and they may ignore other subtypes of CTCs. We used the second-generation CanPatrol™ CTC detection technology to isolate, identify, and classify CTCs. This technology can classify CTCs in peripheral blood into three categories based on the CTC phenotypes and can also detect white blood cells (WBCs). This expands the scope of CTC-related research. Using this platform, we have compared the clinical value of various CTC subtypes in the early prediction of the recurrence of hepatocellular carcinoma and found that the mesenchymal CTC count is a promising marker for predicting the prognosis of hepatocellular carcinoma (3, 4). Later, we found that CTCs in the peripheral blood of patients with hepatocellular carcinoma were occasionally associated with non-malignant cells such as white blood cells (WBCs) and reported that the formation of such CTC-associated WBC (CTC-WBC) clusters indicated a poor prognosis (5). Many studies have shown that epithelial–mesenchymal transition (EMT) plays a key role in tumor recurrence and metastasis, and immune cells may directly or indirectly participate in multiple steps of tumor progression and promote the metastasis of CTCs (6–9). These conclusions stimulated our interest in this research. At present, the specific genetic mechanism of EMT and immune cell-associated CTC cluster formation is still unclear.

The development of CTC enrichment technology and next-generation sequencing (NGS) technology provides a window for exploring this mechanism. Tumor cells transform themselves and interact with the microenvironment to produce heterogeneity so that tumors have a variety of viability (10, 11). Changes in the gene profile of tumor cells determine the heterogeneity of tumors, and it is important to understand the temporal and spatial changes of the genome of these patients longitudinally (12).

Here, we explored the interaction between mesenchymal CTCs, CTC-WBC clusters, and CTC-DNA genomic alterations in patients with hepatocellular carcinoma. We used the CanPatrol™ platform CTC enrichment and analysis technology in combination with NGS. These studies explored the potential uses of the platform. We demonstrated the association between mesenchymal CTCs and CTC-WBC clusters and specific mutation profiles.

## Materials and Methods

### Patients and Peripheral Blood Samples

From January 2016 to December 2019, a total of 29 tissue specimens and peripheral blood specimens from patients with hepatocellular carcinoma who were treated at the Zhujiang Hospital, Southern Medical University were collected. All participants signed informed consent forms. Peripheral blood was collected in a 10 ml EDTA vacuum container, and tumor tissue samples were collected in a specimen bag. All samples were processed within 24 h. This research protocol was approved by the Zhujiang Hospital, Southern Medical University (2021-KY-022-01) and was carried out in accordance with the Declaration of Helsinki.

### Circulating Tumor Cell Enrichment and Detection

**Figure 1:**
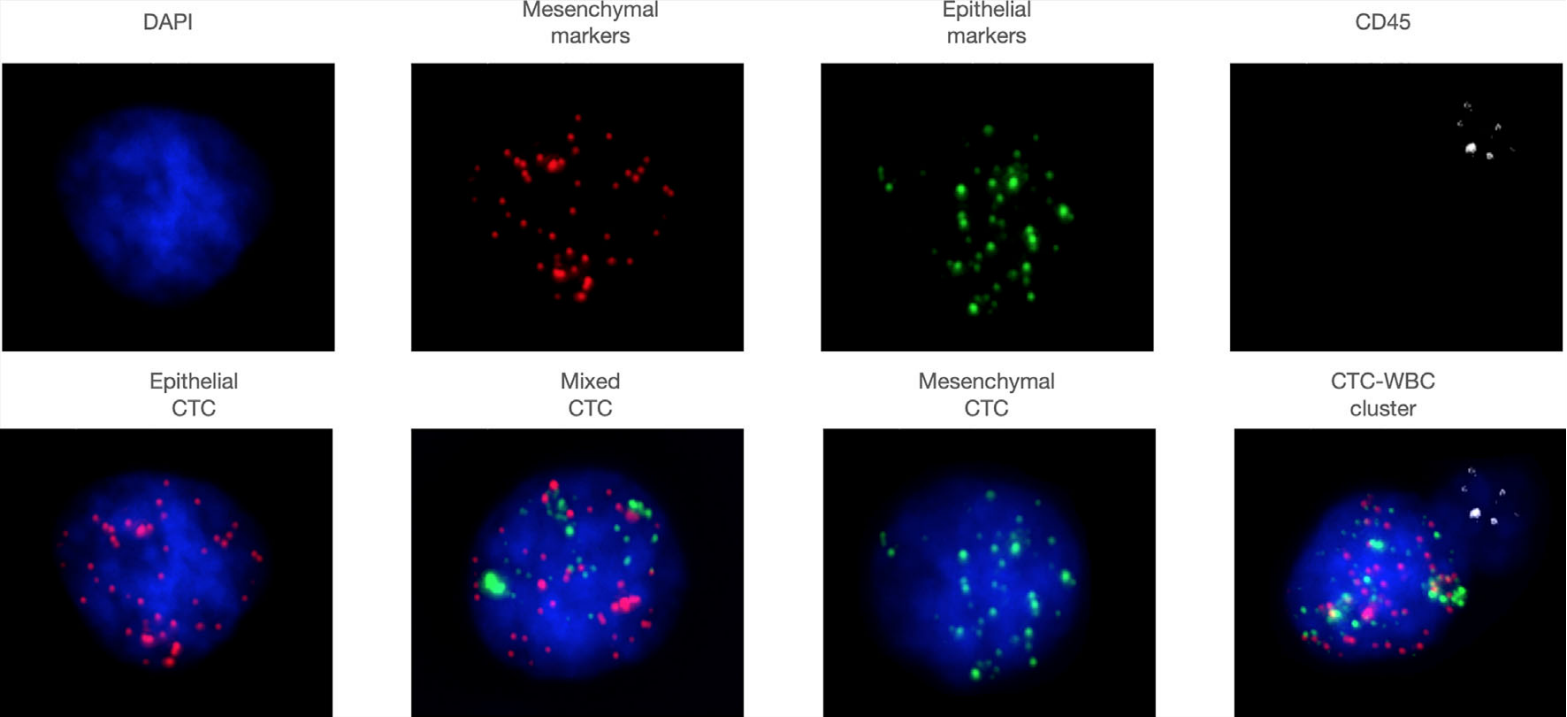
Representative images of various subtypes of CTCs and CTC-WBC clusters. Epithelial cells are positive for EpCAM and CK8/18/19 (red fluorescence), mesenchymal cells are positive for vimentin/twist (green fluorescence), and mixed cells show red fluorescence and green fluorescence. Nuclei were labeled with 40,6-diamidino- 2-phenylindole (DAPI) (blue fluorescence). White blood cells were labeled with CD45 (white fluorescence).

We collected 7.5 ml of peripheral venous blood from the patient, and the CanPatrol™ CTC analysis system (SurExam, China) was used to capture and enrich CTC. RNA-ISH was used to label various CTC subtypes (13, 14). Epithelial cells are positive for EpCAM and CK8/18/19 (red fluorescence); mesenchymal cells are positive for vimentin/twist (green fluorescence), and mixed cells show red fluorescence and green fluorescence. Nuclei were labeled with 40,6-diamidino-2-phenylindole (DAPI) (blue fluorescence). White blood cells were labeled with CD45 (white fluorescence) (**Figure 1**). Fluorescence microscopy was used to analyze the labeled cells

### DNA Extraction and Quantification

DNA from CTCs was prepared immediately after isolation, and whole-genome amplification (WGA) was carried out using the GenomePlex Single Cell Whole Genome Amplification Kit (SIGMA). The concentration of purified DNA from CTCs was detected using the QubitTM dsDNA HA assay Kit (Thermo Fisher Scientific). The DNA extraction steps are as follows:

1) Preparation of cell lysis solution
WGA lysate and Proteinase K (20 mg/ml) are configured in a ratio of 4:1 (each sample needs 28 μl, the minimum configuration is 50 μl) and mix well.
2) Collecting cells
Stick a layer of double-sided tape on a PCR plate and set it aside. Mark the barcode on the prepared 1.5 ml centrifuge tube and paste it upside down on the double-sided tape. Add 14 μl cell lysate to the cap of the 1.5 ml centrifuge tube; the lysate flattens the bottom of the cover. Place the membrane corresponding to the sample on the slide and cut the membrane with a disposable blade. Put the cut membrane on the lid of the 1.5 ml centrifuge tube and add 14 μl cell lysate on the membrane; make sure the membrane is in the cell lysate. After the collection, close the tube cap. Repeat the above steps to complete multi-tube cell collection.
3) Cell disintegration
Invert the 1.5 ml centrifuge tube from which the cells have been collected, place it in the incubator, and incubate overnight with gentle shaking at 56°C. Take out the 1.5 ml centrifuge tube, centrifuge at 10,000×g, and centrifuge for 5 min.
4) Whole genome amplification
Take a new PCR tube, add 2 μl digestion enzyme II to 32 μl genome lysate; mix well for use. Transfer the supernatant in the 1.5 ml tube to a new PCR tube (mark number first); add to the cell lysate 1 μl of the solution prepared in the previous step; mix well. Place it in a PCR machine (cycle 1: 50°C, 30 min; cycle 2: 99°C, 4 min). After 4 min at 99°C, insert into the cooler immediately to cool for 2 min. **Table S3** provides a list of primers to amplify these genes.

### Targeted NGS

Library preparation was performed using the Ion AmpliSeq™ Library Kit (Thermo Fisher Scientific). The 207 amplicons were from 50 oncogenes and tumor suppressor genes (**Additional file 1**: **Table S1**). Sequencing was performed on an Ion Proton System using Ion PI™ Hi-Q™ Chef Kit (Thermo Fisher Scientific).

### Quality Assessment and Mutation Calling

A total of 29 tumor tissue samples and 29 peripheral venous blood samples were collected. However, for one of the blood samples, not enough DNA fragments were detected based on a targeted sequencing coverage greater than 10,000×, and it failed to pass quality control. Finally, DNA was extracted from 29 tumor tissue specimens and 28 CTC specimens for next-generation sequencing analysis. DNA sequenced raw reads QC was then performed using fastp (version 0.20.0) (15). The sequencing coverage was 500× for tissue samples and 1,000× for CTC samples. The clean reads were then aligned to the human hg38 genome using BWA (16), and the resulting BAM files were generated. To identify the variants in the samples, we used Mutect2 (17) for single nucleotide variants (SNVs) and indel calling. Samtools (18) and GATK FilterMutectCalls were then used to extract high-confidence somatic variants from the raw results and to remove clusters of false positives; germline variants were fused in GATK with the gnomAD database. Finally, mutation calls were annotated using ANNOVAR (19).

### Statistical Analysis

Patient characteristics were reported using descriptive analyses. Associations between mesenchymal CTC count or CTC-WBC cluster count and specific genomic alterations were tested using the Mann–Whitney U test. All analyses were performed using IBM SPSS Statistics 25.0. All statistical analyses were two-tailed, and a p-value <0.05 was considered statistically significant.

## Result

### Patient Characteristics

The patient characteristics of the 29 HCC patients are shown in **Table 1**. There were three women and 26 men, with a median age of 54 (range: 25–66) years. Regarding BCLC staging, there were two (6.9%) cases of stage 0, six (20.7%) cases of stage A, 17 (58.6%) cases of stage B, and four (13.8%) cases of stage C. The cutoff values for mesenchymal CTCs and CTC-WBC clusters were determined in our previous studies (3, 5). Mesenchymal CTC and CTC-WBC cluster counts showed 21 (75%) mesenchymal CTCs ≥1 and nine (32.1%) CTC-WBC clusters ≥2.

**Table 1:** Patient characteristics.

Characteristics	n(%)
Gender	
male	26(89.7)
female	3(10.3)
Age [years, median (range)]	54(25–66)
BCLC stage	
0	2(6.9)
A	6(20.7)
B	17(58.6)
C	4(13.8)
Mesenchymal CTC number	
≥1	21(75.0)
<1	7(25.0)
CTC-WBC clusters number	
≥2	9(32.1)
<2	19(67.9)

BCLC, Barcelona Clinical Liver Cancer; CTCs, circulating tumor cells; CTC-WBC, circulating tumor cell associated white blood cells.

### CTC-DNA Genomic Alterations

**Figure 2:**
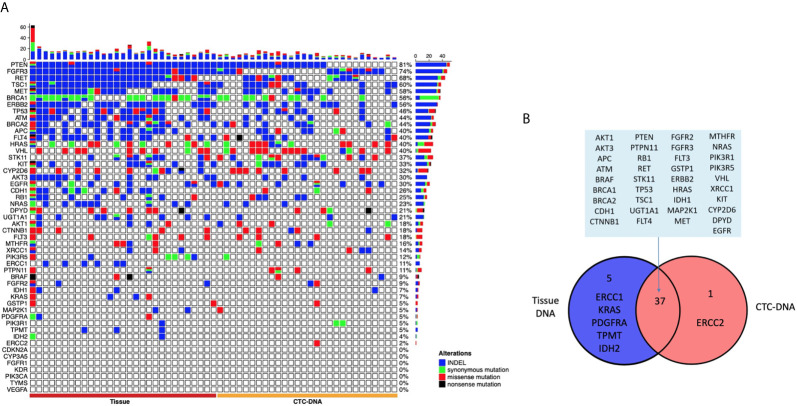
The landscape of genome alteration using tissue and circulating tumor cells (CTCs) for targeted next-generation sequencing. **(A)** The heat map on the left shows the genomic change landscape of the tissue sample, and the right is the genomic change landscape of the CTC sample. The four colors indicate different mutation types, indels (blue), missense mutations (red), synonymous mutations (green), and meaningless mutations (black). **(B)** Venn diagram of genomic alterations using tissue and CTCs for targeted next-generation sequencing.

Among the 29 patients examined, tumor tissue DNA was obtained from all the 29 patients, and CTC-DNA was obtained from 28 patients to confirm the occurrence of somatic mutations. The analysis results of tumor tissue DNA and CTC-DNA mutations are shown in **Figure 2**, and the mutation details are shown in **Table S2**. Targeted sequencing of 207 amplicons and 50 genes was performed. The results of genomic mutation profiling of CTC-DNA and tissue-DNA (**Figure 2A**) suggested that the genetic mutational concordance between profiles of CTC-DNA and tumor tissue DNA was not high. Missense mutations were detected in 34 genes in tumor tissue samples, and missense mutations were detected in 29 genes in CTC samples. Both tumor tissue samples and CTC samples detected 37 identical mutant genes, including PTEN, MET, EGFR, RET, FGFR3, *etc.* ERCC2 was only detected in CTC samples, while ERCC1, KRAS, PDGFRA, TPMT, IDH2 were only detected in tumor tissue samples (**Figure 2B**). Due to the temporal and spatial heterogeneity of tumors, tumor tissue DNA and CTC-DNA have different characteristics. Therefore, combined analysis improves the sensitivity of detection without reducing specificity.

### Correlation Between Mesenchymal CTC Count and CTC-DNA Alterations

**Figure 3:**
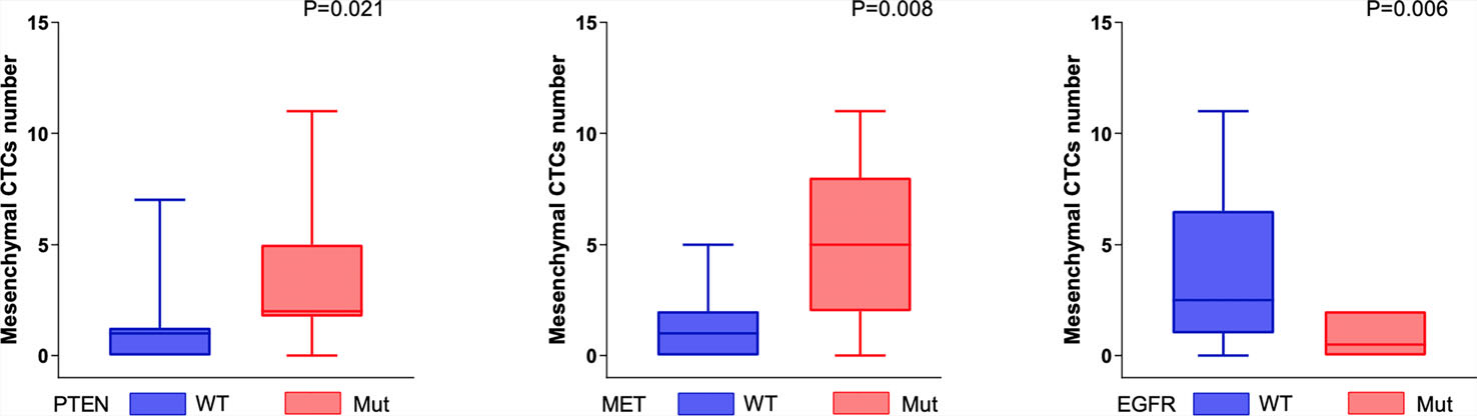
Genomic alterations associated with mesenchymal CTCs. The number of mesenchymal CTCs was positively correlated with the somatic genomic alterations in PTEN and MET genes (PTEN, P = 0.021; MET, P  = 0.008, Mann–Whitney U test), and negatively correlated with the somatic genomic alterations in EGFR (P = 0.006, Mann–Whitney U test).

A lower number of mesenchymal CTCs were detected in the peripheral blood of patients with somatic genomic alterations in the EGFR gene (P < 0.05, Mann–Whitney U test). In contrast, a higher number of mesenchymal CTCs were detected in the peripheral blood of patients with somatic genomic alterations in the PTEN and MET genes (P < 0.05, Mann–Whitney U test) (**Figure 3**).

### Correlation Between CTC-WBC Cluster Count and CTC-DNA Alterations

**Figure 4:**
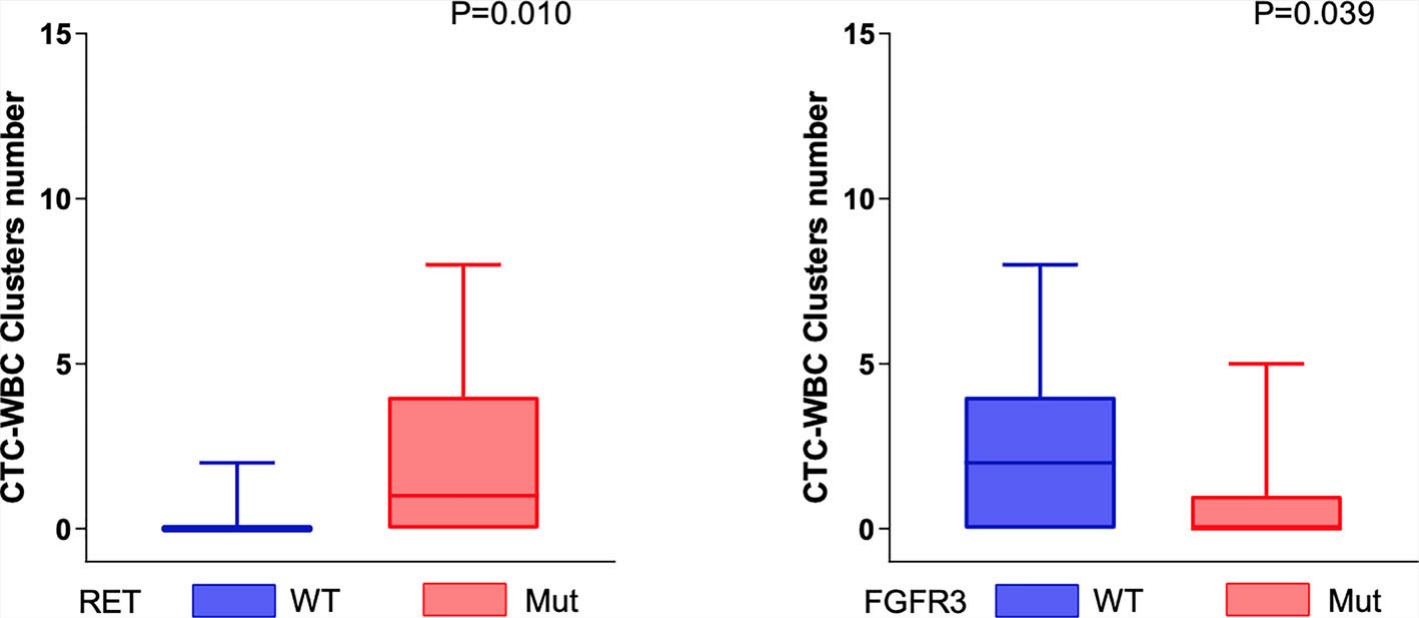
Genomic alterations associated with mesenchymal CTC-WBC clusters. The number of CTC-WBC clusters was positively correlated with the somatic genomic alterations in RET genes (P = 0.01, Mann–Whitney U test) and negatively correlated with the somatic genomic alterations in FGFR3 (P = 0.039, Mann–Whitney U test).

The CTC-WBC clusters in the peripheral blood of patients were significantly positively correlated with the somatic genomic alterations in the RET gene and were significantly negatively correlated with the somatic genomic alterations in the FGFR3 gene (all P < 0.05, Mann–Whitney U test) (**Figure 4**).

### Case Vignette

We monitored the genomic alterations in circulating tumor cells and mesenchymal CTC and CTC-WBC cluster counts in four patients during treatment. All the four patients underwent radical surgery, recurrence occurred after surgery, and their disease progressed after treatment. They received individualized radiofrequency ablation (RFA), transcatheter arterial chemoembolization (TACE), chemotherapy, targeted therapy, or immunotherapy depending on their condition. We monitored the genomic alterations in circulating tumor cells and mesenchymal CTC and CTC-WBC cluster counts before and after surgery, before and after chemotherapy, before and after targeted therapy, and before and after immunotherapy (**Figure S1A**). Representative baseline and disease progression CT images are shown in **Figure S1B**. By monitoring the genomic alterations in circulating tumor cells, we found that the tumor showed obvious heterogeneity in the treatment process. Such minimally invasive methods could enhance the understanding of the mechanisms underlying cancer diversity and drug resistance. The number of DNA mutation genes in four patients decreased after surgical treatment, and this trend continues after the disease progresses. However, the mesenchymal CTC and CTC-WBC cluster counts showed a significant increase after the progression. There was no correlation between the number of gene mutations and the mesenchymal CTC or CTC-WBC cluster counts. Genomic alterations in circulating tumor cells can be used to monitor the heterogeneity that occurs before and after tumor treatment, and the mesenchymal CTC and CTC-WBC cluster counts can be used to monitor disease progression. This liquid biopsy technique has potential clinical applications.

## Discussion

The superiority of liquid biopsy technology in understanding the temporal and spatial heterogeneity of tumors is gradually being accepted. In this study, we used the CTC analysis technology of the CanPatrol™ platform to effectively capture mesenchymal CTC and CTC-WBC clusters from the peripheral blood of hepatocellular carcinoma patients and used NGS technology to perform targeted sequencing of CTC-DNA. We evaluated the relationship between genomic alterations in circulating tumor cells and mesenchymal CTC and CTC-WBC clusters. In addition, we designed a strategy that combines CTC counts and analysis of genomic alterations in circulating tumor cells to monitor changes in patients throughout the course of treatment, hoping to guide clinical practice.

In this study, when comparing the mutations detected in the tumor tissues of patients with hepatocellular carcinoma, we found that in some blood samples, mutations not found in the tumor tissue DNA can be detected in CTC-DNA. The gene mutational spectra of the two specimens are not completely consistent. The different characteristics of tumor tissue DNA and CTC-DNA may be due to the temporal and spatial heterogeneity of tumors. Although many studies have confirmed the feasibility of capturing CTC based on the Canpatrol™ platform (13, 14), we still cannot rule out that technical reasons cause this difference to be affected by the analyzed tumor percentage. The method of capturing CTCs to extract DNA and sequencing can compensate for the deficiency of tumor tissue DNA. Compared to tissue DNA taken from the local tumor, CTC-DNA comes from different parts of the primary tumor or different metastases. CTC-DNA is the molecular representative of the tumor as a whole (20). CTC-DNA can better reflect the temporal and spatial heterogeneity of tumors. In addition, liquid biopsy is more convenient for obtaining clinical specimens and has better clinical application potential.

We observed that the number of mesenchymal CTCs in peripheral blood is positively correlated with mutations in the PTEN and MET genes, and negatively correlated with mutations in the FGFR3 gene. PTEN is a tumor suppressor gene for a variety of tumors. Studies on prostate cancer have found that the loss of the prostate epithelial PTEN gene leads to the transformation of pluripotent progenitor cells and epithelial-to-mesenchymal transition, which is related to the invasion ability of prostate cancer (21). Another study on glioblastoma found that stable inactivation of EGFR induced a mesenchymal to epithelioid transition in EGFR-amplified xenografts, thereby affecting the aggressiveness of tumor cells (22). Our findings further support these conclusions. This provides a direction for further analysis of the mechanism of mesenchymal cell formation at the genetic level. However, the role of PTEN and FGFR3 gene mutations in the EMT process in hepatocellular carcinoma still needs further analysis.

In addition, mutation of the RET gene is associated with a large number of CTC-WBC clusters, while mutation of the FGFR3 gene is associated with a small number of CTC-WBC clusters. The RET gene is a member of the cadherin superfamily and plays a role in cell adhesion, chemical signaling, and cell movement, all of which may contribute to the metastasis process. A study on bladder cancer found that the FGFR3 gene is related to cell adhesion. Mutation of the FGFR3 gene is beneficial in the development and progression of bladder cancer since it changes a key gene that regulates the cell–cell and cell–matrix adhesion properties of urothelial cells (23). The mechanism of CTC-WBC cluster formation is very complicated, and there are various reasons. Among them, the influence of cell adhesion is one of the important reasons (24, 25). A study on the formation mechanism of peripheral blood CTC-WBC clusters in breast cancer patients suggested that plasma plakoglobin is directly related to E-cadherin, which interacts and plays a vital role between desmosomal cadherin and the intermediate filament cytoskeletons (26). Our previous study found that the formation of CTC-WBC clusters in the peripheral blood of patients with hepatocellular carcinoma is a biomarker of poor prognosis (5). However, are certain gene mutations in the peripheral circulation the original driving force for the combination of soil (immune cells) and seeds (tumor cells)? The specific mechanism of this “oil and seed” formation still needs to be explored.

This study had some limitations. First, our study consisted of a small number of samples. Second, the selection based on 50 genes to be tested is controversial. In summary, we optimized the efficiency of capturing CTC platforms and revealed the importance of CTC and CTC-DNA as diagnostic tools or monitoring methods. Reflecting the temporal and spatial heterogeneity of tumors, disease monitoring has obvious clinical potential. Further analysis allows us to understand the different biological characteristics of the mesenchymal CTC and CTC-WBC clusters based on specific genomic changes. It lays the foundation for the next steps in CTC-related research.

## Data Availability Statement

The datasets presented in this study can be found in online repositories. The names of the repository/repositories and accession number(s) can be found below: NCBI and accession PRJNA719251 https://dataview.ncbi.nlm.nih.gov/object/PRJNA719251?reviewer=4bmf2fqki2thmrc4e3li77ih5l.

## Ethics Statement

The research protocol has been approved by the Institutional Review Committee of Zhujiang Hospital, Southern Medical University (2021-KY-022-01).

## Author Contributions

Conception and design: MP. Acquisition of data: CZ, HL, and KC. Analysis and interpretation of data: CW. Writing, review and/or revision of the manuscript: QL. Analysis of bioinformatics: WH. Administrative, technical, or material support: MP. Study supervision: MP. All authors contributed to the article and approved the submitted version.

## Funding

This research was supported by a grant from the National Natural Science Foundation of China (no.82072627) and Natural Science Foundation of Guangdong Province of China (no.2016A030313626).

## Conflict of Interest

The authors declare that the research was conducted in the absence of any commercial or financial relationships that could be construed as a potential conflict of interest.

## Supplementary Material

Supplementary Figure 1
**(A, B)** Case vignette monitoring the genomic alterations in circulating tumor cells and mesenchymal CTC and CTC-WBC cluster counts.
