# Supplementary material for: Correlation Between Circulating Tumor Cell DNA Genomic Alterations and Mesenchymal CTCs or CTC-Associated White Blood Cell Clusters in Hepatocellular Carcinoma
Source: Front Oncol. 2021 Jun 11;11:686365. doi: 10.3389/fonc.2021.686365 (PMC8226125; doi:10.3389/fonc.2021.686365)
Supplement: Supplementary file 1 [file Table_1.docx]

**Table S1:** Customized Specificly 50-gene panel (Surexam)

| AKT1 | AKT3 | APC | ATM | BRAF | BRCA1 | BRCA2 | CDH1 | CDKN2A | CTNNB1 |
| --- | --- | --- | --- | --- | --- | --- | --- | --- | --- |
| CYP2D6 | CYP3A5 | DPYD | EGFR | ERCC1 | ERCC2 | FGFR1 | FGFR2 | FGFR3 | FLT3 |
| GSTP1 | ERBB2 | HRAS | IDH1 | IDH2 | KIT | KRAS | MAP2K1 | MET | MTHFR |
| NRAS | PDGFRA | PIK3CA | PIK3R1 | PIK3R5 | PTEN | PTPN11 | RB1 | RET | STK11 |
| TP53 | TPMT | TSC1 | TYMS | UGT1A1 | VEGFA | KDR | FLT4 | VHL | XRCC1 |
