# Supplementary material for: Correlation Between Circulating Tumor Cell DNA Genomic Alterations and Mesenchymal CTCs or CTC-Associated White Blood Cell Clusters in Hepatocellular Carcinoma
Source: Front Oncol. 2021 Jun 11;11:686365. doi: 10.3389/fonc.2021.686365 (PMC8226125; doi:10.3389/fonc.2021.686365)
Supplement: Supplementary file 2 [file Table_2.docx]

Table S2. The mutation profiles of CTC-DNA and Tissue-DNA

| Patient No. | Sample types | Chromesome | Gene | Alterations | Nucleotide change |  |
| --- | --- | --- | --- | --- | --- | --- |
| 1 | ctc | chr1 | DPYD | nonsynonymous SNV | exon13:c.A1627G |  |
| 1 | ctc | chr1 | NRAS | frameshift deletion | exon3:c.251_252del |  |
| 1 | ctc | chr4 | FGFR3 | frameshift deletion | exon9:c.1200delC |  |
| 1 | ctc | chr7 | EGFR | frameshift insertion | exon14:c.1512dupC |  |
| 1 | ctc | chr7 | MET | synonymous SNV | exon2:c.A564T |  |
| 1 | ctc | chr9 | TSC1 | frameshift deletion | exon14:c.1369delT |  |
| 1 | ctc | chr10 | RET | frameshift deletion | exon16:c.2768delT |  |
| 1 | ctc | chr10 | PTEN | frameshift deletion | exon8:c.963delA |  |
| 1 | ctc | chr11 | HRAS | nonsynonymous SNV | exon3:c.C248G |  |
| 1 | ctc | chr13 | FLT3 | nonsynonymous SNV | exon14:c.A1763G |  |
| 1 | ctc | chr13 | FLT3 | nonsynonymous SNV | exon11:c.G1386T |  |
| 1 | ctc | chr13 | BRCA2 | frameshift insertion | exon11:c.5578dupA |  |
| 1 | ctc | chr17 | PIK3R5 | frameshift deletion | exon2:c.13delG |  |
| 1 | ctc | chr17 | BRCA1 | synonymous SNV | exon1:c.A30G |  |
| 1 | ctc | chr19 | STK11 | frameshift deletion | exon4:c.571delA |  |
| 1 | tissue | chr1 | AKT3 | frameshift deletion | exon5:c.516delA |  |
| 1 | tissue | chr3 | CTNNB1 | nonsynonymous SNV | exon3:c.A95G |  |
| 1 | tissue | chr4 | FGFR3 | frameshift deletion | exon9:c.1200delC |  |
| 1 | tissue | chr4 | KIT | frameshift deletion | exon17:c.2430delT |  |
| 1 | tissue | chr5 | APC | frameshift deletion | exon7:c.818delT |  |
| 1 | tissue | chr5 | FLT4 | frameshift deletion | exon11:c.1500delC |  |
| 1 | tissue | chr7 | MET | frameshift deletion | exon2:c.1138delA |  |
| 1 | tissue | chr7 | MET | frameshift deletion | exon18:c.2428delA |  |
| 1 | tissue | chr9 | TSC1 | frameshift deletion | exon14:c.1369delT |  |
| 1 | tissue | chr10 | PTEN | frameshift deletion | exon8:c.963delA |  |
| 1 | tissue | chr10 | PTEN | frameshift deletion | exon8:c.1009delT |  |
| 1 | tissue | chr13 | BRCA2 | frameshift deletion | exon11:c.5930delT |  |
| 1 | tissue | chr13 | RB1 | frameshift deletion | exon20:c.2048delT |  |
| 1 | tissue | chr14 | AKT1 | frameshift deletion | exon3:c.49delG |  |
| 1 | tissue | chr16 | CDH1 | frameshift deletion | exon3:c.204delT |  |
| 1 | tissue | chr17 | ERBB2 | frameshift deletion | exon21:c.2578delA |  |
| 1 | tissue | chr17 | BRCA1 | frameshift deletion | exon18:c.5061delT |  |
| 1 | tissue | chr17 | BRCA1 | synonymous SNV | exon1:c.A30G |  |
| 1 | tissue | chr1 | DPYD | nonsynonymous SNV | exon22:c.T2834C |  |
| 1 | tissue | chr2 | IDH1 | frameshift deletion | exon4:c.347delA |  |
| 1 | tissue | chr2 | UGT1A1 | frameshift deletion | exon1:c.296delT |  |
| 1 | tissue | chr3 | VHL | nonsynonymous SNV | exon1:c.G332A |  |
| 1 | tissue | chr3 | CTNNB1 | nonsynonymous SNV | exon3:c.A95G |  |
| 1 | tissue | chr4 | FGFR3 | frameshift deletion | exon9:c.1200delC |  |
| 1 | tissue | chr5 | APC | frameshift deletion | exon7:c.818delT |  |
| 1 | tissue | chr5 | APC | frameshift deletion | exon14:c.2486delA |  |
| 1 | tissue | chr5 | FLT4 | frameshift deletion | exon30:c.3976delG |  |
| 1 | tissue | chr10 | PTEN | frameshift deletion | exon8:c.963delA |  |
| 1 | tissue | chr11 | ATM | frameshift deletion | exon17:c.2479delA |  |
| 1 | tissue | chr17 | ERBB2 | frameshift deletion | exon21:c.2578delA |  |
| 1 | tissue | chr17 | BRCA1 | synonymous SNV | exon1:c.A30G |  |
| 1 | tissue | chr3 | CTNNB1 | nonsynonymous SNV | exon3:c.A95G |  |
| 1 | tissue | chr4 | FGFR3 | frameshift deletion | exon9:c.1200delC |  |
| 1 | tissue | chr4 | PDGFRA | frameshift deletion | exon18:c.2487delA |  |
| 1 | tissue | chr5 | APC | frameshift deletion | exon7:c.818delT |  |
| 1 | tissue | chr5 | APC | frameshift deletion | exon14:c.2486delA |  |
| 1 | tissue | chr10 | RET | frameshift deletion | exon16:c.2768delT |  |
| 1 | tissue | chr10 | PTEN | frameshift deletion | exon8:c.963delA |  |
| 1 | tissue | chr11 | ATM | frameshift deletion | exon17:c.2479delA |  |
| 1 | tissue | chr13 | BRCA2 | frameshift deletion | exon11:c.6125delA |  |
| 1 | tissue | chr17 | TP53 | frameshift deletion | exon4:c.506delC |  |
| 1 | tissue | chr17 | ERBB2 | frameshift deletion | exon21:c.2578delA |  |
| 1 | tissue | chr17 | BRCA1 | frameshift deletion | exon1:c.61delA |  |
| 2 | ctc | chr2 | UGT1A1 | frameshift insertion | exon1:c.273dupT |  |
| 2 | ctc | chr4 | FGFR3 | nonsynonymous SNV | exon9:c.A1211G |  |
| 2 | ctc | chr4 | KIT | frameshift insertion | exon10:c.1552dupC |  |
| 2 | ctc | chr5 | FLT4 | stopgain | exon11:c.G1521A |  |
| 2 | ctc | chr7 | EGFR | frameshift insertion | exon14:c.1512dupC |  |
| 2 | ctc | chr10 | PTEN | frameshift deletion | exon8:c.963delA |  |
| 2 | ctc | chr13 | FLT3 | nonsynonymous SNV | exon14:c.A1763G |  |
| 2 | ctc | chr14 | AKT1 | nonsynonymous SNV | exon3:c.T103A |  |
| 2 | ctc | chr22 | CYP2D6 | nonsynonymous SNV | exon1:c.C100T |  |
| 2 | tissue | chr1 | DPYD | nonsynonymous SNV | exon22:c.T2834C |  |
| 2 | tissue | chr2 | UGT1A1 | frameshift deletion | exon1:c.296delT |  |
| 2 | tissue | chr4 | FGFR3 | frameshift deletion | exon9:c.1200delC |  |
| 2 | tissue | chr7 | MET | frameshift deletion | exon2:c.1025delT |  |
| 2 | tissue | chr7 | MET | frameshift deletion | exon18:c.2428delA |  |
| 2 | tissue | chr9 | TSC1 | frameshift deletion | exon14:c.1743delA |  |
| 2 | tissue | chr10 | RET | frameshift deletion | exon16:c.2768delT |  |
| 2 | tissue | chr10 | PTEN | frameshift deletion | exon8:c.963delA |  |
| 2 | tissue | chr11 | ATM | frameshift deletion | exon17:c.2479delA |  |
| 2 | tissue | chr16 | CDH1 | frameshift insertion | exon6:c.715dupG |  |
| 2 | tissue | chr17 | TP53 | nonsynonymous SNV | exon3:c.G351T |  |
| 2 | tissue | chr17 | ERBB2 | frameshift deletion | exon21:c.2578delA |  |
| 3 | tissue | chr1 | AKT3 | frameshift deletion | exon5:c.516delA |  |
| 3 | tissue | chr3 | VHL | nonsynonymous SNV | exon1:c.G332A |  |
| 3 | tissue | chr4 | KIT | frameshift deletion | exon17:c.2430delT |  |
| 3 | tissue | chr5 | APC | frameshift deletion | exon7:c.818delT |  |
| 3 | tissue | chr5 | FLT4 | frameshift deletion | exon30:c.3976delG |  |
| 3 | tissue | chr9 | TSC1 | frameshift deletion | exon14:c.1369delT |  |
| 3 | tissue | chr10 | RET | frameshift deletion | exon16:c.2768delT |  |
| 3 | tissue | chr10 | PTEN | frameshift deletion | exon8:c.963delA |  |
| 3 | tissue | chr11 | HRAS | nonsynonymous SNV | exon2:c.C5T |  |
| 3 | tissue | chr13 | BRCA2 | frameshift deletion | exon11:c.6125delA |  |
| 3 | tissue | chr13 | RB1 | frameshift deletion | exon20:c.2048delT |  |
| 3 | tissue | chr16 | CDH1 | nonsynonymous SNV | exon6:c.G769T |  |
| 3 | tissue | chr17 | TP53 | frameshift insertion | exon4:c.484dupG |  |
| 3 | tissue | chr17 | TP53 | frameshift deletion | exon2:c.173delC |  |
| 3 | tissue | chr17 | ERBB2 | frameshift insertion | exon20:c.2336dupC |  |
| 3 | tissue | chr17 | BRCA1 | synonymous SNV | exon1:c.A30G |  |
| 4 | ctc | chr1 | NRAS | frameshift deletion | exon4:c.359delT |  |
| 4 | ctc | chr2 | UGT1A1 | frameshift deletion | exon1:c.296delT |  |
| 4 | ctc | chr3 | VHL | synonymous SNV | exon1:c.G252A |  |
| 4 | ctc | chr4 | FGFR3 | frameshift deletion | exon9:c.1200delC |  |
| 4 | ctc | chr5 | FLT4 | nonsynonymous SNV | exon11:c.A1477G |  |
| 4 | ctc | chr7 | EGFR | frameshift insertion | exon14:c.1512dupC |  |
| 4 | ctc | chr10 | RET | frameshift deletion | exon16:c.2768delT |  |
| 4 | ctc | chr10 | PTEN | frameshift deletion | exon8:c.963delA |  |
| 4 | ctc | chr11 | HRAS | synonymous SNV | exon2:c.T81C |  |
| 4 | ctc | chr13 | BRCA2 | frameshift deletion | exon11:c.5646delA |  |
| 4 | ctc | chr15 | MAP2K1 | frameshift insertion | exon2:c.222dupG |  |
| 4 | ctc | chr17 | PIK3R5 | synonymous SNV | exon2:c.G18A |  |
| 4 | ctc | chr17 | BRCA1 | synonymous SNV | exon1:c.A30G |  |
| 4 | tissue | chr1 | MTHFR | synonymous SNV | exon5:c.C795T |  |
| 4 | tissue | chr1 | DPYD | synonymous SNV | exon13:c.T1635C |  |
| 4 | tissue | chr1 | NRAS | synonymous SNV | exon3:c.C285T |  |
| 4 | tissue | chr1 | AKT3 | frameshift deletion | exon5:c.516delA |  |
| 4 | tissue | chr2 | IDH1 | nonsynonymous SNV | exon4:c.G373A |  |
| 4 | tissue | chr2 | IDH1 | nonsynonymous SNV | exon4:c.G328A |  |
| 4 | tissue | chr3 | CTNNB1 | nonsynonymous SNV | exon3:c.T97G |  |
| 4 | tissue | chr3 | CTNNB1 | nonsynonymous SNV | exon3:c.C130T |  |
| 4 | tissue | chr4 | PDGFRA | synonymous SNV | exon15:c.A2040G |  |
| 4 | tissue | chr4 | KIT | stopgain | exon2:c.G198A |  |
| 4 | tissue | chr4 | KIT | stopgain | exon10:c.C1543T |  |
| 4 | tissue | chr5 | APC | frameshift deletion | exon7:c.818delT |  |
| 4 | tissue | chr5 | APC | frameshift deletion | exon14:c.2486delA |  |
| 4 | tissue | chr5 | APC | synonymous SNV | exon14:c.C2532T |  |
| 4 | tissue | chr5 | APC | frameshift deletion | exon14:c.4444delT |  |
| 4 | tissue | chr5 | APC | synonymous SNV | exon14:c.G4473A |  |
| 4 | tissue | chr5 | APC | nonsynonymous SNV | exon14:c.G4679A |  |
| 4 | tissue | chr5 | FLT4 | frameshift deletion | exon30:c.3976delG |  |
| 4 | tissue | chr7 | EGFR | nonsynonymous SNV | exon12:c.T1274C |  |
| 4 | tissue | chr7 | EGFR | nonsynonymous SNV | exon13:c.G1405A |  |
| 4 | tissue | chr7 | MET | nonsynonymous SNV | exon2:c.G572A |  |
| 4 | tissue | chr7 | MET | nonsynonymous SNV | exon13:c.C1628T |  |
| 4 | tissue | chr7 | MET | nonsynonymous SNV | exon18:c.G2419A |  |
| 4 | tissue | chr7 | BRAF | stopgain | exon11:c.G1349A |  |
| 4 | tissue | chr9 | TSC1 | nonsynonymous SNV | exon14:c.C1456T |  |
| 4 | tissue | chr9 | TSC1 | nonsynonymous SNV | exon14:c.G1373A |  |
| 4 | tissue | chr9 | TSC1 | frameshift deletion | exon6:c.455delT |  |
| 4 | tissue | chr10 | PTEN | frameshift deletion | exon9:c.1044delA |  |
| 4 | tissue | chr10 | PTEN | synonymous SNV | exon9:c.T1110C |  |
| 4 | tissue | chr10 | FGFR2 | frameshift deletion | exon11:c.1574delA |  |
| 4 | tissue | chr10 | FGFR2 | nonsynonymous SNV | exon2:c.C335T |  |
| 4 | tissue | chr11 | HRAS | synonymous SNV | exon3:c.G237A |  |
| 4 | tissue | chr11 | HRAS | frameshift deletion | exon2:c.84delT |  |
| 4 | tissue | chr12 | KRAS | nonsynonymous SNV | exon3:c.C149T |  |
| 4 | tissue | chr13 | FLT3 | nonsynonymous SNV | exon16:c.C2051T |  |
| 4 | tissue | chr13 | BRCA2 | nonsynonymous SNV | exon11:c.T5894C |  |
| 4 | tissue | chr13 | RB1 | nonsynonymous SNV | exon17:c.G1674A |  |
| 4 | tissue | chr14 | AKT1 | nonsynonymous SNV | exon3:c.G49A |  |
| 4 | tissue | chr15 | IDH2 | synonymous SNV | exon2:c.C111T |  |
| 4 | tissue | chr16 | CDH1 | nonsynonymous SNV | exon8:c.A1120G |  |
| 4 | tissue | chr17 | TP53 | synonymous SNV | exon4:c.G501A |  |
| 4 | tissue | chr17 | TP53 | nonsynonymous SNV | exon3:c.G351T |  |
| 4 | tissue | chr17 | TP53 | synonymous SNV | exon1:c.C90T |  |
| 4 | tissue | chr17 | PIK3R5 | nonsynonymous SNV | exon2:c.G3A |  |
| 4 | tissue | chr1 | MTHFR | frameshift deletion | exon8:c.1390delG |  |
| 4 | tissue | chr1 | DPYD | nonsynonymous SNV | exon13:c.C1642T |  |
| 4 | tissue | chr1 | NRAS | synonymous SNV | exon3:c.C213T |  |
| 4 | tissue | chr3 | CTNNB1 | nonsynonymous SNV | exon3:c.G199A |  |
| 4 | tissue | chr4 | FGFR3 | frameshift deletion | exon9:c.1200delC |  |
| 4 | tissue | chr4 | KIT | nonsynonymous SNV | exon2:c.G94A |  |
| 4 | tissue | chr4 | KIT | nonsynonymous SNV | exon9:c.A1537G |  |
| 4 | tissue | chr4 | KIT | synonymous SNV | exon17:c.T2403C |  |
| 4 | tissue | chr5 | APC | frameshift deletion | exon7:c.818delT |  |
| 4 | tissue | chr5 | APC | nonsynonymous SNV | exon14:c.G3913A |  |
| 4 | tissue | chr5 | FLT4 | stopgain | exon30:c.C3988T |  |
| 4 | tissue | chr5 | FLT4 | frameshift deletion | exon30:c.3976delG |  |
| 4 | tissue | chr5 | FLT4 | nonsynonymous SNV | exon11:c.G1534A |  |
| 4 | tissue | chr5 | FLT4 | nonsynonymous SNV | exon11:c.C1517T |  |
| 4 | tissue | chr7 | EGFR | nonsynonymous SNV | exon14:c.C1547T |  |
| 4 | tissue | chr7 | MET | stopgain | exon2:c.C493T |  |
| 4 | tissue | chr7 | MET | synonymous SNV | exon18:c.G2367A |  |
| 4 | tissue | chr7 | MET | frameshift deletion | exon18:c.2428delA |  |
| 4 | tissue | chr9 | TSC1 | synonymous SNV | exon14:c.G1755A |  |
| 4 | tissue | chr9 | TSC1 | nonsynonymous SNV | exon4:c.T134G |  |
| 4 | tissue | chr10 | PTEN | frameshift deletion | exon8:c.963delA |  |
| 4 | tissue | chr11 | HRAS | nonsynonymous SNV | exon3:c.C202T |  |
| 4 | tissue | chr11 | GSTP1 | nonsynonymous SNV | exon5:c.A313G |  |
| 4 | tissue | chr11 | ATM | nonsynonymous SNV | exon63:c.G8995A |  |
| 4 | tissue | chr12 | PTPN11 | nonsynonymous SNV | exon3:c.A164G |  |
| 4 | tissue | chr12 | PTPN11 | nonsynonymous SNV | exon13:c.A1487G |  |
| 4 | tissue | chr12 | PTPN11 | nonsynonymous SNV | exon13:c.A1531G |  |
| 4 | tissue | chr13 | BRCA2 | nonsynonymous SNV | exon11:c.A5581G |  |
| 4 | tissue | chr13 | RB1 | synonymous SNV | exon17:c.A1668G |  |
| 4 | tissue | chr14 | AKT1 | synonymous SNV | exon3:c.G48A |  |
| 4 | tissue | chr16 | CDH1 | nonsynonymous SNV | exon3:c.C182T |  |
| 4 | tissue | chr16 | CDH1 | synonymous SNV | exon8:c.T1050C |  |
| 4 | tissue | chr17 | TP53 | nonsynonymous SNV | exon3:c.C284T |  |
| 4 | tissue | chr17 | TP53 | stopgain | exon1:c.C10T |  |
| 4 | tissue | chr17 | ERBB2 | nonsynonymous SNV | exon21:c.A2549G |  |
| 4 | tissue | chr19 | XRCC1 | nonsynonymous SNV | exon6:c.C580T |  |
| 4 | tissue | chr19 | XRCC1 | frameshift deletion | exon6:c.565delG |  |
| 4 | tissue | chr22 | CYP2D6 | nonsynonymous SNV | exon1:c.C100T |  |
| 4 | tissue | chr4 | FGFR3 | frameshift deletion | exon9:c.1200delC |  |
| 4 | tissue | chr5 | FLT4 | frameshift deletion | exon30:c.3976delG |  |
| 4 | tissue | chr7 | EGFR | synonymous SNV | exon14:c.G1560A |  |
| 4 | tissue | chr7 | MET | frameshift deletion | exon2:c.1025delT |  |
| 4 | tissue | chr7 | MET | frameshift deletion | exon18:c.2351delA |  |
| 4 | tissue | chr7 | MET | frameshift deletion | exon18:c.2428delA |  |
| 4 | tissue | chr9 | TSC1 | frameshift deletion | exon14:c.1743delA |  |
| 4 | tissue | chr9 | TSC1 | frameshift deletion | exon14:c.1369delT |  |
| 4 | tissue | chr10 | RET | frameshift deletion | exon16:c.2768delT |  |
| 4 | tissue | chr10 | PTEN | frameshift deletion | exon1:c.37delA |  |
| 4 | tissue | chr10 | PTEN | nonsynonymous SNV | exon5:c.G395A |  |
| 4 | tissue | chr10 | PTEN | frameshift deletion | exon8:c.963delA |  |
| 4 | tissue | chr11 | ATM | frameshift deletion | exon17:c.2479delA |  |
| 4 | tissue | chr13 | RB1 | frameshift deletion | exon17:c.1581delT |  |
| 4 | tissue | chr16 | CDH1 | synonymous SNV | exon11:c.T1665C |  |
| 4 | tissue | chr17 | ERBB2 | frameshift deletion | exon21:c.2578delA |  |
| 4 | tissue | chr17 | BRCA1 | synonymous SNV | exon1:c.A30G |  |
| 5 | ctc | chr4 | FGFR3 | frameshift deletion | exon9:c.1200delC |  |
| 5 | ctc | chr7 | EGFR | nonsynonymous SNV | exon14:c.C1503G |  |
| 5 | ctc | chr7 | EGFR | frameshift insertion | exon14:c.1512dupC |  |
| 5 | ctc | chr10 | RET | nonsynonymous SNV | exon11:c.T1928C |  |
| 5 | ctc | chr10 | PTEN | frameshift deletion | exon8:c.963delA |  |
| 5 | ctc | chr11 | HRAS | nonsynonymous SNV | exon3:c.G183T |  |
| 5 | ctc | chr13 | RB1 | nonsynonymous SNV | exon20:c.T2036C |  |
| 5 | ctc | chr17 | PIK3R5 | frameshift deletion | exon2:c.8delC |  |
| 5 | ctc | chr17 | ERBB2 | frameshift insertion | exon20:c.2336dupC |  |
| 5 | tissue | chr1 | NRAS | frameshift deletion | exon4:c.359delT |  |
| 5 | tissue | chr1 | NRAS | frameshift deletion | exon2:c.49delA |  |
| 5 | tissue | chr1 | AKT3 | frameshift deletion | exon5:c.516delA |  |
| 5 | tissue | chr3 | VHL | nonsynonymous SNV | exon1:c.G332A |  |
| 5 | tissue | chr4 | FGFR3 | frameshift deletion | exon9:c.1200delC |  |
| 5 | tissue | chr5 | APC | frameshift deletion | exon7:c.818delT |  |
| 5 | tissue | chr5 | FLT4 | frameshift deletion | exon30:c.3976delG |  |
| 5 | tissue | chr7 | MET | frameshift deletion | exon2:c.488delC |  |
| 5 | tissue | chr7 | MET | frameshift deletion | exon2:c.1138delA |  |
| 5 | tissue | chr9 | TSC1 | frameshift deletion | exon14:c.1369delT |  |
| 5 | tissue | chr10 | RET | frameshift deletion | exon16:c.2768delT |  |
| 5 | tissue | chr10 | PTEN | frameshift deletion | exon8:c.963delA |  |
| 5 | tissue | chr13 | BRCA2 | frameshift deletion | exon11:c.5930delT |  |
| 5 | tissue | chr13 | BRCA2 | frameshift deletion | exon11:c.6133delT |  |
| 5 | tissue | chr16 | CDH1 | frameshift deletion | exon6:c.743delT |  |
| 5 | tissue | chr17 | TP53 | frameshift deletion | exon4:c.506delC |  |
| 5 | tissue | chr17 | ERBB2 | frameshift deletion | exon21:c.2578delA |  |
| 5 | tissue | chr17 | BRCA1 | frameshift deletion | exon1:c.61delA |  |
| 5 | tissue | chr17 | BRCA1 | synonymous SNV | exon1:c.A30G |  |
| 5 | tissue | chr19 | ERCC1 | frameshift deletion | exon3:c.326delG |  |
| 6 | ctc | chr4 | FGFR3 | frameshift deletion | exon9:c.1200delC |  |
| 6 | ctc | chr7 | MET | frameshift deletion | exon2:c.1138delA |  |
| 6 | ctc | chr10 | RET | frameshift deletion | exon11:c.1991delC |  |
| 6 | ctc | chr10 | RET | synonymous SNV | exon11:c.C1992A |  |
| 6 | ctc | chr10 | PTEN | frameshift deletion | exon8:c.963delA |  |
| 6 | ctc | chr11 | HRAS | nonsynonymous SNV | exon2:c.C5T |  |
| 6 | ctc | chr13 | BRCA2 | frameshift deletion | exon11:c.5578delA |  |
| 6 | ctc | chr13 | BRCA2 | nonsynonymous SNV | exon11:c.A5659G |  |
| 6 | ctc | chr14 | AKT1 | nonsynonymous SNV | exon3:c.A141C |  |
| 6 | ctc | chr17 | PIK3R5 | synonymous SNV | exon2:c.A21C |  |
| 6 | ctc | chr17 | BRCA1 | synonymous SNV | exon1:c.A30G |  |
| 6 | ctc | chr22 | CYP2D6 | frameshift insertion | exon1:c.112dupC |  |
| 6 | tissue | chr3 | VHL | nonsynonymous SNV | exon1:c.G332A |  |
| 6 | tissue | chr5 | FLT4 | frameshift deletion | exon30:c.3976delG |  |
| 6 | tissue | chr9 | TSC1 | frameshift deletion | exon6:c.455delT |  |
| 6 | tissue | chr10 | RET | frameshift deletion | exon16:c.2768delT |  |
| 6 | tissue | chr10 | PTEN | frameshift deletion | exon8:c.963delA |  |
| 6 | tissue | chr11 | ATM | frameshift deletion | exon17:c.2479delA |  |
| 6 | tissue | chr13 | BRCA2 | frameshift deletion | exon11:c.5930delT |  |
| 6 | tissue | chr16 | CDH1 | synonymous SNV | exon8:c.G1020A |  |
| 6 | tissue | chr17 | TP53 | frameshift deletion | exon4:c.506delC |  |
| 6 | tissue | chr1 | NRAS | frameshift deletion | exon2:c.49delA |  |
| 6 | tissue | chr3 | VHL | nonsynonymous SNV | exon1:c.G332A |  |
| 6 | tissue | chr4 | FGFR3 | frameshift deletion | exon9:c.1200delC |  |
| 6 | tissue | chr6 | TPMT | frameshift deletion | exon8:c.663delT |  |
| 6 | tissue | chr7 | MET | frameshift deletion | exon2:c.1025delT |  |
| 6 | tissue | chr7 | MET | frameshift deletion | exon2:c.1138delA |  |
| 6 | tissue | chr7 | MET | frameshift deletion | exon18:c.2351delA |  |
| 6 | tissue | chr7 | MET | frameshift deletion | exon18:c.2428delA |  |
| 6 | tissue | chr9 | TSC1 | frameshift deletion | exon14:c.1369delT |  |
| 6 | tissue | chr10 | RET | frameshift deletion | exon16:c.2768delT |  |
| 6 | tissue | chr10 | PTEN | frameshift deletion | exon8:c.963delA |  |
| 6 | tissue | chr16 | CDH1 | synonymous SNV | exon8:c.G1020A |  |
| 6 | tissue | chr17 | BRCA1 | frameshift deletion | exon1:c.61delA |  |
| 6 | tissue | chr17 | BRCA1 | synonymous SNV | exon1:c.A30G |  |
| 6 | tissue | chr19 | ERCC1 | frameshift deletion | exon3:c.326delG |  |
| 7 | ctc | chr7 | EGFR | nonsynonymous SNV | exon13:c.A1469G |  |
| 7 | ctc | chr7 | EGFR | frameshift insertion | exon14:c.1512dupC |  |
| 7 | ctc | chr7 | EGFR | synonymous SNV | exon15:c.C1776T |  |
| 7 | ctc | chr7 | MET | frameshift deletion | exon2:c.1138delA |  |
| 7 | ctc | chr10 | PTEN | frameshift deletion | exon9:c.1044delA |  |
| 7 | ctc | chr10 | FGFR2 | nonsynonymous SNV | exon2:c.G346A |  |
| 7 | ctc | chr11 | GSTP1 | nonsynonymous SNV | exon5:c.G252C |  |
| 7 | ctc | chr13 | FLT3 | nonsynonymous SNV | exon14:c.A1763G |  |
| 7 | ctc | chr16 | CDH1 | synonymous SNV | exon11:c.A1686G |  |
| 7 | ctc | chr19 | STK11 | nonsynonymous SNV | exon6:c.A797G |  |
| 7 | ctc | chr19 | STK11 | frameshift deletion | exon6:c.837delC |  |
| 7 | ctc | chr19 | STK11 | nonsynonymous SNV | exon8:c.G998A |  |
| 7 | ctc | chr19 | ERCC2 | nonsynonymous SNV | exon23:c.T2267C |  |
| 7 | tissue | chr1 | DPYD | nonsynonymous SNV | exon22:c.T2834C |  |
| 7 | tissue | chr4 | FGFR3 | frameshift deletion | exon9:c.1200delC |  |
| 7 | tissue | chr7 | EGFR | frameshift deletion | exon12:c.1336delA |  |
| 7 | tissue | chr9 | TSC1 | frameshift deletion | exon14:c.1743delA |  |
| 7 | tissue | chr10 | RET | frameshift deletion | exon16:c.2768delT |  |
| 7 | tissue | chr10 | PTEN | frameshift deletion | exon8:c.963delA |  |
| 7 | tissue | chr13 | BRCA2 | frameshift deletion | exon11:c.5930delT |  |
| 7 | tissue | chr17 | ERBB2 | frameshift deletion | exon21:c.2578delA |  |
| 7 | tissue | chr17 | BRCA1 | synonymous SNV | exon1:c.A30G |  |
| 7 | tissue | chr19 | STK11 | nonsynonymous SNV | exon1:c.G154A |  |
| 7 | tissue | chr19 | XRCC1 | frameshift deletion | exon6:c.565delG |  |
| 7 | tissue | chr22 | CYP2D6 | nonsynonymous SNV | exon1:c.C100T |  |
| 7 | tissue | chr1 | MTHFR | frameshift deletion | exon5:c.729delC |  |
| 7 | tissue | chr1 | AKT3 | frameshift deletion | exon5:c.516delA |  |
| 7 | tissue | chr2 | UGT1A1 | frameshift deletion | exon1:c.296delT |  |
| 7 | tissue | chr3 | VHL | nonsynonymous SNV | exon1:c.G332A |  |
| 7 | tissue | chr4 | FGFR3 | frameshift deletion | exon9:c.1200delC |  |
| 7 | tissue | chr4 | KIT | frameshift deletion | exon17:c.2430delT |  |
| 7 | tissue | chr5 | APC | frameshift deletion | exon7:c.818delT |  |
| 7 | tissue | chr5 | FLT4 | frameshift deletion | exon30:c.3976delG |  |
| 7 | tissue | chr7 | BRAF | stopgain | exon15:c.1774delA |  |
| 7 | tissue | chr10 | RET | frameshift deletion | exon16:c.2768delT |  |
| 7 | tissue | chr10 | PTEN | frameshift deletion | exon8:c.963delA |  |
| 7 | tissue | chr11 | HRAS | nonsynonymous SNV | exon2:c.C5T |  |
| 7 | tissue | chr11 | ATM | frameshift deletion | exon17:c.2479delA |  |
| 7 | tissue | chr13 | BRCA2 | nonsynonymous SNV | exon11:c.G5924A |  |
| 7 | tissue | chr13 | BRCA2 | frameshift deletion | exon11:c.5930delT |  |
| 7 | tissue | chr14 | AKT1 | frameshift deletion | exon3:c.49delG |  |
| 7 | tissue | chr16 | CDH1 | frameshift deletion | exon6:c.743delT |  |
| 7 | tissue | chr16 | CDH1 | frameshift deletion | exon11:c.1714delG |  |
| 7 | tissue | chr17 | TP53 | frameshift deletion | exon4:c.506delC |  |
| 7 | tissue | chr17 | TP53 | frameshift insertion | exon4:c.484dupG |  |
| 7 | tissue | chr17 | ERBB2 | frameshift deletion | exon21:c.2578delA |  |
| 7 | tissue | chr19 | STK11 | frameshift deletion | exon6:c.837delC |  |
| 7 | tissue | chr22 | CYP2D6 | nonsynonymous SNV | exon1:c.C100T |  |
| 8 | ctc | chr1 | NRAS | frameshift deletion | exon4:c.359delT |  |
| 8 | ctc | chr4 | FGFR3 | nonsynonymous SNV | exon9:c.T1250C |  |
| 8 | ctc | chr4 | KIT | frameshift insertion | exon10:c.1552dupC |  |
| 8 | ctc | chr5 | APC | frameshift insertion | exon14:c.3872dupA |  |
| 8 | ctc | chr7 | MET | frameshift deletion | exon18:c.2428delA |  |
| 8 | ctc | chr10 | RET | nonsynonymous SNV | exon11:c.T1937A |  |
| 8 | ctc | chr10 | PTEN | frameshift deletion | exon8:c.963delA |  |
| 8 | ctc | chr17 | PIK3R5 | synonymous SNV | exon2:c.T45C |  |
| 8 | ctc | chr17 | BRCA1 | nonsynonymous SNV | exon1:c.A29G |  |
| 8 | tissue | chr1 | AKT3 | frameshift deletion | exon5:c.516delA |  |
| 8 | tissue | chr3 | CTNNB1 | nonsynonymous SNV | exon3:c.G94A |  |
| 8 | tissue | chr3 | CTNNB1 | nonsynonymous SNV | exon3:c.G101A |  |
| 8 | tissue | chr4 | FGFR3 | frameshift deletion | exon9:c.1200delC |  |
| 8 | tissue | chr7 | EGFR | frameshift deletion | exon12:c.1336delA |  |
| 8 | tissue | chr7 | MET | frameshift deletion | exon18:c.2428delA |  |
| 8 | tissue | chr10 | RET | frameshift deletion | exon16:c.2768delT |  |
| 8 | tissue | chr10 | PTEN | frameshift deletion | exon8:c.963delA |  |
| 8 | tissue | chr17 | ERBB2 | frameshift deletion | exon21:c.2578delA |  |
| 8 | tissue | chr17 | ERBB2 | frameshift deletion | exon21:c.2641delG |  |
| 8 | tissue | chr17 | BRCA1 | synonymous SNV | exon1:c.A30G |  |
| 8 | tissue | chr22 | CYP2D6 | nonsynonymous SNV | exon1:c.C100T |  |
| 8 | tissue | chr2 | UGT1A1 | frameshift deletion | exon1:c.296delT |  |
| 8 | tissue | chr4 | FGFR3 | frameshift deletion | exon9:c.1200delC |  |
| 8 | tissue | chr7 | EGFR | frameshift deletion | exon12:c.1336delA |  |
| 8 | tissue | chr9 | TSC1 | frameshift deletion | exon14:c.1743delA |  |
| 8 | tissue | chr10 | RET | frameshift deletion | exon16:c.2768delT |  |
| 8 | tissue | chr10 | PTEN | frameshift deletion | exon8:c.963delA |  |
| 8 | tissue | chr10 | FGFR2 | frameshift deletion | exon11:c.1574delA |  |
| 8 | tissue | chr11 | ATM | frameshift deletion | exon17:c.2479delA |  |
| 8 | tissue | chr17 | TP53 | frameshift deletion | exon4:c.506delC |  |
| 8 | tissue | chr17 | TP53 | frameshift deletion | exon1:c.7delT |  |
| 8 | tissue | chr17 | ERBB2 | frameshift deletion | exon21:c.2578delA |  |
| 8 | tissue | chr17 | ERBB2 | frameshift deletion | exon21:c.2641delG |  |
| 8 | tissue | chr22 | CYP2D6 | nonsynonymous SNV | exon1:c.C100T |  |
| 9 | ctc | chr1 | MTHFR | synonymous SNV | exon8:c.T1404C |  |
| 9 | ctc | chr3 | VHL | nonsynonymous SNV | exon1:c.A329T |  |
| 9 | ctc | chr3 | CTNNB1 | nonsynonymous SNV | exon3:c.A191G |  |
| 9 | ctc | chr4 | KIT | synonymous SNV | exon17:c.C2410A |  |
| 9 | ctc | chr5 | APC | frameshift deletion | exon14:c.2486delA |  |
| 9 | ctc | chr5 | APC | frameshift deletion | exon14:c.4328delA |  |
| 9 | ctc | chr7 | MET | frameshift deletion | exon18:c.2428delA |  |
| 9 | ctc | chr9 | TSC1 | frameshift deletion | exon14:c.1369delT |  |
| 9 | ctc | chr10 | RET | nonsynonymous SNV | exon11:c.G1921A |  |
| 9 | ctc | chr10 | PTEN | frameshift deletion | exon8:c.963delA |  |
| 9 | ctc | chr11 | HRAS | nonsynonymous SNV | exon2:c.G88A |  |
| 9 | ctc | chr11 | ATM | frameshift deletion | exon17:c.2589delT |  |
| 9 | ctc | chr13 | FLT3 | nonsynonymous SNV | exon14:c.A1763G |  |
| 9 | ctc | chr13 | RB1 | frameshift insertion | exon17:c.1641dupA |  |
| 9 | ctc | chr17 | ERBB2 | frameshift deletion | exon21:c.2578delA |  |
| 9 | ctc | chr19 | STK11 | synonymous SNV | exon1:c.G234A |  |
| 9 | ctc | chr19 | STK11 | synonymous SNV | exon4:c.G495A |  |
| 9 | ctc | chr19 | XRCC1 | nonsynonymous SNV | exon6:c.A539G |  |
| 9 | ctc | chr22 | CYP2D6 | nonsynonymous SNV | exon5:c.C733T |  |
| 9 | tissue | chr1 | NRAS | frameshift deletion | exon4:c.384delA |  |
| 9 | tissue | chr3 | CTNNB1 | nonsynonymous SNV | exon3:c.A95G |  |
| 9 | tissue | chr7 | MET | frameshift deletion | exon2:c.1025delT |  |
| 9 | tissue | chr7 | MET | frameshift deletion | exon2:c.1138delA |  |
| 9 | tissue | chr9 | TSC1 | frameshift deletion | exon14:c.1369delT |  |
| 9 | tissue | chr10 | RET | frameshift deletion | exon16:c.2768delT |  |
| 9 | tissue | chr10 | PTEN | frameshift deletion | exon8:c.963delA |  |
| 9 | tissue | chr11 | ATM | frameshift deletion | exon17:c.2479delA |  |
| 9 | tissue | chr13 | BRCA2 | frameshift deletion | exon11:c.5930delT |  |
| 9 | tissue | chr17 | ERBB2 | frameshift deletion | exon21:c.2578delA |  |
| 9 | tissue | chr17 | BRCA1 | synonymous SNV | exon1:c.A30G |  |
| 9 | tissue | chr22 | CYP2D6 | nonsynonymous SNV | exon1:c.C100T |  |
| 10 | ctc | chr4 | KIT | frameshift insertion | exon10:c.1552dupC |  |
| 10 | ctc | chr5 | FLT4 | frameshift deletion | exon30:c.3976delG |  |
| 10 | ctc | chr9 | TSC1 | nonsynonymous SNV | exon14:c.T1382C |  |
| 10 | ctc | chr10 | RET | synonymous SNV | exon11:c.A2013G |  |
| 10 | ctc | chr10 | RET | frameshift deletion | exon16:c.2768delT |  |
| 10 | ctc | chr10 | PTEN | frameshift deletion | exon8:c.963delA |  |
| 10 | ctc | chr11 | HRAS | nonframeshift substitution | exon3:c.251_252CT,HRAS | |
| 10 | ctc | chr12 | PTPN11 | nonsynonymous SNV | exon13:c.T1553C |  |
| 10 | ctc | chr14 | AKT1 | nonsynonymous SNV | exon3:c.T56G |  |
| 10 | ctc | chr17 | BRCA1 | nonsynonymous SNV | exon1:c.A47G |  |
| 10 | ctc | chr19 | STK11 | nonsynonymous SNV | exon4:c.A532G |  |
| 10 | ctc | chr19 | STK11 | synonymous SNV | exon8:c.C1062T |  |
| 10 | tissue | chr4 | FGFR3 | frameshift deletion | exon9:c.1200delC |  |
| 10 | tissue | chr5 | APC | frameshift deletion | exon7:c.818delT |  |
| 10 | tissue | chr7 | MET | frameshift deletion | exon2:c.1138delA |  |
| 10 | tissue | chr9 | TSC1 | frameshift deletion | exon14:c.1743delA |  |
| 10 | tissue | chr9 | TSC1 | frameshift deletion | exon11:c.999delA |  |
| 10 | tissue | chr10 | PTEN | frameshift deletion | exon8:c.963delA |  |
| 10 | tissue | chr11 | ATM | frameshift deletion | exon17:c.2479delA |  |
| 10 | tissue | chr13 | RB1 | frameshift deletion | exon20:c.2048delT |  |
| 10 | tissue | chr17 | TP53 | frameshift deletion | exon1:c.7delT |  |
| 10 | tissue | chr17 | ERBB2 | frameshift deletion | exon21:c.2578delA |  |
| 10 | tissue | chr1 | NRAS | frameshift deletion | exon2:c.49delA |  |
| 10 | tissue | chr1 | AKT3 | frameshift deletion | exon5:c.516delA |  |
| 10 | tissue | chr7 | MET | frameshift deletion | exon2:c.1138delA |  |
| 10 | tissue | chr9 | TSC1 | frameshift deletion | exon14:c.1743delA |  |
| 10 | tissue | chr9 | TSC1 | frameshift deletion | exon14:c.1369delT |  |
| 10 | tissue | chr9 | TSC1 | frameshift deletion | exon11:c.999delA |  |
| 10 | tissue | chr10 | RET | frameshift deletion | exon16:c.2768delT |  |
| 10 | tissue | chr10 | PTEN | frameshift deletion | exon8:c.963delA |  |
| 10 | tissue | chr11 | ATM | frameshift deletion | exon17:c.2479delA |  |
| 10 | tissue | chr13 | BRCA2 | frameshift deletion | exon11:c.6125delA |  |
| 10 | tissue | chr16 | CDH1 | frameshift deletion | exon3:c.168delT |  |
| 10 | tissue | chr16 | CDH1 | frameshift deletion | exon6:c.743delT |  |
| 10 | tissue | chr17 | BRCA1 | synonymous SNV | exon1:c.A30G |  |
| 10 | tissue | chr19 | ERCC1 | frameshift deletion | exon3:c.326delG |  |
| 11 | ctc | chr3 | VHL | nonsynonymous SNV | exon1:c.A329T |  |
| 11 | ctc | chr5 | APC | frameshift insertion | exon14:c.3867dupA |  |
| 11 | ctc | chr5 | FLT4 | frameshift deletion | exon30:c.3976delG |  |
| 11 | ctc | chr7 | MET | frameshift deletion | exon2:c.505delC |  |
| 11 | ctc | chr9 | TSC1 | frameshift deletion | exon14:c.1743delA |  |
| 11 | ctc | chr11 | HRAS | nonsynonymous SNV | exon3:c.T242C |  |
| 11 | ctc | chr13 | FLT3 | nonsynonymous SNV | exon14:c.A1763G |  |
| 11 | ctc | chr13 | BRCA2 | synonymous SNV | exon11:c.A5688C |  |
| 11 | ctc | chr14 | AKT1 | synonymous SNV | exon3:c.C87T |  |
| 11 | tissue | chr1 | AKT3 | frameshift deletion | exon5:c.516delA |  |
| 11 | tissue | chr4 | FGFR3 | frameshift deletion | exon9:c.1200delC |  |
| 11 | tissue | chr4 | KIT | frameshift deletion | exon17:c.2430delT |  |
| 11 | tissue | chr5 | FLT4 | frameshift deletion | exon30:c.3976delG |  |
| 11 | tissue | chr7 | EGFR | frameshift deletion | exon12:c.1336delA |  |
| 11 | tissue | chr7 | MET | frameshift deletion | exon2:c.488delC |  |
| 11 | tissue | chr7 | MET | frameshift deletion | exon2:c.1138delA |  |
| 11 | tissue | chr9 | TSC1 | frameshift deletion | exon14:c.1743delA |  |
| 11 | tissue | chr9 | TSC1 | frameshift deletion | exon14:c.1369delT |  |
| 11 | tissue | chr9 | TSC1 | frameshift deletion | exon11:c.999delA |  |
| 11 | tissue | chr10 | PTEN | frameshift deletion | exon8:c.963delA |  |
| 11 | tissue | chr10 | PTEN | frameshift deletion | exon9:c.1044delA |  |
| 11 | tissue | chr11 | GSTP1 | nonsynonymous SNV | exon5:c.A313G |  |
| 11 | tissue | chr13 | BRCA2 | frameshift deletion | exon11:c.5578delA |  |
| 11 | tissue | chr17 | ERBB2 | frameshift deletion | exon21:c.2578delA |  |
| 12 | ctc | chr1 | DPYD | nonsynonymous SNV | exon22:c.T2834C |  |
| 12 | ctc | chr4 | KIT | frameshift insertion | exon9:c.1509dupT |  |
| 12 | ctc | chr10 | PTEN | frameshift deletion | exon8:c.963delA |  |
| 12 | ctc | chr17 | ERBB2 | frameshift deletion | exon21:c.2578delA |  |
| 12 | ctc | chr17 | BRCA1 | synonymous SNV | exon1:c.A30G |  |
| 12 | ctc | chr19 | STK11 | synonymous SNV | exon6:c.G843T |  |
| 12 | tissue | chr1 | DPYD | nonsynonymous SNV | exon22:c.T2834C |  |
| 12 | tissue | chr1 | NRAS | synonymous SNV | exon3:c.G168A |  |
| 12 | tissue | chr4 | FGFR3 | frameshift deletion | exon9:c.1200delC |  |
| 12 | tissue | chr4 | PDGFRA | nonsynonymous SNV | exon14:c.G1944A |  |
| 12 | tissue | chr4 | KIT | nonsynonymous SNV | exon2:c.G173A |  |
| 12 | tissue | chr4 | KIT | nonsynonymous SNV | exon13:c.G1885A |  |
| 12 | tissue | chr4 | KIT | synonymous SNV | exon17:c.C2394T |  |
| 12 | tissue | chr5 | APC | synonymous SNV | exon14:c.G3924A |  |
| 12 | tissue | chr9 | TSC1 | frameshift deletion | exon11:c.999delA |  |
| 12 | tissue | chr10 | PTEN | frameshift deletion | exon8:c.963delA |  |
| 12 | tissue | chr10 | PTEN | nonsynonymous SNV | exon9:c.C1069T |  |
| 12 | tissue | chr11 | ATM | nonsynonymous SNV | exon17:c.G2467A |  |
| 12 | tissue | chr11 | ATM | synonymous SNV | exon17:c.G2544A |  |
| 12 | tissue | chr12 | KRAS | nonsynonymous SNV | exon4:c.G340A |  |
| 12 | tissue | chr12 | PTPN11 | nonsynonymous SNV | exon3:c.G190A |  |
| 12 | tissue | chr12 | PTPN11 | frameshift deletion | exon13:c.1486delA |  |
| 12 | tissue | chr12 | PTPN11 | synonymous SNV | exon13:c.G1530A |  |
| 12 | tissue | chr13 | FLT3 | synonymous SNV | exon11:c.G1350A |  |
| 12 | tissue | chr13 | BRCA2 | frameshift deletion | exon11:c.5646delA |  |
| 12 | tissue | chr13 | BRCA2 | nonsynonymous SNV | exon11:c.A5900T |  |
| 12 | tissue | chr13 | RB1 | frameshift deletion | exon17:c.1581delT |  |
| 12 | tissue | chr13 | RB1 | synonymous SNV | exon20:c.G1983A |  |
| 12 | tissue | chr19 | STK11 | synonymous SNV | exon8:c.G1023A |  |
| 12 | tissue | chr1 | NRAS | frameshift deletion | exon4:c.359delT |  |
| 12 | tissue | chr1 | AKT3 | frameshift deletion | exon5:c.516delA |  |
| 12 | tissue | chr4 | FGFR3 | frameshift deletion | exon9:c.1200delC |  |
| 12 | tissue | chr4 | KIT | frameshift deletion | exon17:c.2430delT |  |
| 12 | tissue | chr5 | APC | frameshift deletion | exon14:c.2486delA |  |
| 12 | tissue | chr5 | FLT4 | frameshift deletion | exon30:c.3976delG |  |
| 12 | tissue | chr5 | FLT4 | frameshift deletion | exon11:c.1500delC |  |
| 12 | tissue | chr9 | TSC1 | frameshift deletion | exon14:c.1743delA |  |
| 12 | tissue | chr9 | TSC1 | frameshift deletion | exon14:c.1369delT |  |
| 12 | tissue | chr9 | TSC1 | frameshift deletion | exon11:c.999delA |  |
| 12 | tissue | chr10 | RET | frameshift deletion | exon16:c.2768delT |  |
| 12 | tissue | chr10 | PTEN | frameshift deletion | exon8:c.963delA |  |
| 12 | tissue | chr13 | BRCA2 | frameshift deletion | exon11:c.5930delT |  |
| 12 | tissue | chr14 | AKT1 | frameshift deletion | exon3:c.49delG |  |
| 12 | tissue | chr16 | CDH1 | frameshift deletion | exon6:c.743delT |  |
| 12 | tissue | chr17 | TP53 | frameshift deletion | exon4:c.506delC |  |
| 12 | tissue | chr17 | ERBB2 | frameshift deletion | exon21:c.2578delA |  |
| 12 | tissue | chr19 | STK11 | frameshift deletion | exon4:c.571delA |  |
| 12 | tissue | chr19 | STK11 | frameshift deletion | exon6:c.837delC |  |
| 13 | ctc | chr1 | DPYD | stopgain | exon13:c.1692dupT |  |
| 13 | ctc | chr4 | FGFR3 | frameshift deletion | exon9:c.1200delC |  |
| 13 | ctc | chr10 | RET | nonsynonymous SNV | exon11:c.C1938A |  |
| 13 | ctc | chr10 | PTEN | frameshift deletion | exon8:c.963delA |  |
| 13 | ctc | chr11 | ATM | frameshift deletion | exon17:c.2588delA |  |
| 13 | ctc | chr13 | FLT3 | nonsynonymous SNV | exon14:c.A1763G |  |
| 13 | ctc | chr17 | BRCA1 | synonymous SNV | exon1:c.A30G |  |
| 13 | ctc | chr19 | XRCC1 | frameshift insertion | exon6:c.548dupA |  |
| 13 | ctc | chr22 | CYP2D6 | stopgain | exon4:c.C595T |  |
| 13 | ctc | chr22 | CYP2D6 | frameshift insertion | exon1:c.104dupC |  |
| 13 | tissue | chr4 | KIT | frameshift insertion | exon10:c.1552dupC |  |
| 13 | tissue | chr9 | TSC1 | frameshift deletion | exon11:c.999delA |  |
| 13 | tissue | chr10 | PTEN | frameshift deletion | exon8:c.963delA |  |
| 13 | tissue | chr17 | ERBB2 | frameshift deletion | exon21:c.2578delA |  |
| 13 | tissue | chr17 | BRCA1 | frameshift deletion | exon1:c.61delA |  |
| 13 | tissue | chr17 | BRCA1 | synonymous SNV | exon1:c.A30G |  |
| 13 | tissue | chr22 | CYP2D6 | frameshift insertion | exon1:c.104dupC |  |
| 13 | tissue | chr3 | VHL | nonsynonymous SNV | exon1:c.G332A |  |
| 13 | tissue | chr4 | FGFR3 | frameshift deletion | exon9:c.1200delC |  |
| 13 | tissue | chr5 | FLT4 | frameshift deletion | exon30:c.3976delG |  |
| 13 | tissue | chr7 | MET | frameshift deletion | exon2:c.1025delT |  |
| 13 | tissue | chr7 | MET | frameshift deletion | exon2:c.1138delA |  |
| 13 | tissue | chr7 | MET | frameshift deletion | exon18:c.2428delA |  |
| 13 | tissue | chr9 | TSC1 | frameshift deletion | exon14:c.1369delT |  |
| 13 | tissue | chr10 | PTEN | frameshift deletion | exon8:c.963delA |  |
| 13 | tissue | chr11 | ATM | frameshift deletion | exon17:c.2479delA |  |
| 13 | tissue | chr17 | TP53 | frameshift deletion | exon1:c.7delT |  |
| 13 | tissue | chr17 | ERBB2 | frameshift deletion | exon21:c.2578delA |  |
| 13 | tissue | chr17 | BRCA1 | synonymous SNV | exon1:c.A30G |  |
| 13 | tissue | chr19 | STK11 | frameshift deletion | exon6:c.837delC |  |
| 14 | ctc | chr3 | VHL | nonsynonymous SNV | exon1:c.A329T |  |
| 14 | ctc | chr3 | CTNNB1 | frameshift insertion | exon3:c.199_200insACAACACACGC | |
| 14 | ctc | chr10 | RET | nonframeshift deletion | exon11:c.1905_1907delCAC | |
| 14 | ctc | chr10 | PTEN | frameshift deletion | exon8:c.963delA |  |
| 14 | ctc | chr11 | HRAS | synonymous SNV | exon3:c.C222T |  |
| 14 | ctc | chr11 | ATM | frameshift deletion | exon17:c.2479delA |  |
| 14 | ctc | chr17 | BRCA1 | synonymous SNV | exon1:c.A30G |  |
| 14 | tissue | chr1 | MTHFR | frameshift deletion | exon8:c.1390delG |  |
| 14 | tissue | chr5 | APC | frameshift insertion | exon14:c.4255dupA |  |
| 14 | tissue | chr7 | MET | frameshift deletion | exon2:c.1025delT |  |
| 14 | tissue | chr7 | MET | frameshift deletion | exon2:c.1138delA |  |
| 14 | tissue | chr7 | MET | frameshift deletion | exon18:c.2428delA |  |
| 14 | tissue | chr10 | PTEN | frameshift deletion | exon8:c.963delA |  |
| 14 | tissue | chr10 | FGFR2 | frameshift deletion | exon11:c.1574delA |  |
| 14 | tissue | chr11 | ATM | frameshift deletion | exon17:c.2479delA |  |
| 14 | tissue | chr13 | BRCA2 | frameshift deletion | exon11:c.5578delA |  |
| 14 | tissue | chr13 | BRCA2 | frameshift deletion | exon11:c.5930delT |  |
| 14 | tissue | chr13 | BRCA2 | synonymous SNV | exon11:c.A6111G |  |
| 14 | tissue | chr17 | BRCA1 | frameshift deletion | exon1:c.61delA |  |
| 14 | tissue | chr19 | STK11 | frameshift deletion | exon6:c.837delC |  |
| 15 | ctc | chr3 | VHL | nonsynonymous SNV | exon1:c.C289T |  |
| 15 | ctc | chr3 | VHL | nonsynonymous SNV | exon1:c.A329T |  |
| 15 | ctc | chr7 | MET | frameshift deletion | exon2:c.1138delA |  |
| 15 | ctc | chr9 | TSC1 | frameshift deletion | exon14:c.1743delA |  |
| 15 | ctc | chr10 | RET | frameshift deletion | exon16:c.2768delT |  |
| 15 | ctc | chr10 | PTEN | frameshift deletion | exon8:c.963delA |  |
| 15 | ctc | chr11 | HRAS | nonsynonymous SNV | exon2:c.C5T |  |
| 15 | ctc | chr13 | RB1 | nonsynonymous SNV | exon20:c.G2067T |  |
| 15 | ctc | chr17 | BRCA1 | synonymous SNV | exon1:c.A30G |  |
| 15 | tissue | chr1 | AKT3 | frameshift deletion | exon5:c.516delA |  |
| 15 | tissue | chr4 | FGFR3 | frameshift deletion | exon9:c.1200delC |  |
| 15 | tissue | chr4 | KIT | frameshift deletion | exon17:c.2430delT |  |
| 15 | tissue | chr5 | APC | frameshift deletion | exon7:c.818delT |  |
| 15 | tissue | chr5 | FLT4 | frameshift deletion | exon30:c.3976delG |  |
| 15 | tissue | chr7 | MET | frameshift deletion | exon2:c.488delC |  |
| 15 | tissue | chr9 | TSC1 | synonymous SNV | exon14:c.C1443T |  |
| 15 | tissue | chr9 | TSC1 | frameshift deletion | exon14:c.1369delT |  |
| 15 | tissue | chr10 | RET | frameshift deletion | exon16:c.2768delT |  |
| 15 | tissue | chr10 | PTEN | frameshift deletion | exon8:c.963delA |  |
| 15 | tissue | chr11 | HRAS | synonymous SNV | exon2:c.T81C |  |
| 15 | tissue | chr11 | ATM | frameshift deletion | exon17:c.2479delA |  |
| 15 | tissue | chr17 | TP53 | nonsynonymous SNV | exon3:c.G334C |  |
| 15 | tissue | chr17 | ERBB2 | frameshift deletion | exon21:c.2578delA |  |
| 15 | tissue | chr17 | BRCA1 | synonymous SNV | exon1:c.A30G |  |
| 16 | ctc | chr2 | IDH1 | frameshift insertion | exon4:c.355dupC |  |
| 16 | ctc | chr4 | FGFR3 | frameshift deletion | exon9:c.1200delC |  |
| 16 | ctc | chr11 | HRAS | nonsynonymous SNV | exon2:c.G29A |  |
| 16 | ctc | chr19 | STK11 | nonsynonymous SNV | exon6:c.A775C |  |
| 16 | tissue | chr2 | UGT1A1 | frameshift deletion | exon1:c.296delT |  |
| 16 | tissue | chr4 | FGFR3 | frameshift deletion | exon9:c.1200delC |  |
| 16 | tissue | chr5 | PIK3R1 | frameshift deletion | exon3:c.55delA |  |
| 16 | tissue | chr6 | TPMT | frameshift deletion | exon8:c.663delT |  |
| 16 | tissue | chr7 | MET | frameshift deletion | exon2:c.1138delA |  |
| 16 | tissue | chr7 | MET | frameshift deletion | exon18:c.2428delA |  |
| 16 | tissue | chr10 | PTEN | frameshift deletion | exon8:c.963delA |  |
| 16 | tissue | chr11 | ATM | frameshift deletion | exon17:c.2479delA |  |
| 16 | tissue | chr13 | RB1 | frameshift deletion | exon20:c.2048delT |  |
| 16 | tissue | chr15 | IDH2 | frameshift deletion | exon2:c.77delA |  |
| 16 | tissue | chr17 | BRCA1 | synonymous SNV | exon1:c.A30G |  |
| 16 | tissue | chr22 | CYP2D6 | nonsynonymous SNV | exon1:c.C100T |  |
| 16 | tissue | chr4 | FGFR3 | frameshift deletion | exon9:c.1200delC |  |
| 16 | tissue | chr6 | TPMT | frameshift deletion | exon8:c.663delT |  |
| 16 | tissue | chr7 | MET | frameshift deletion | exon2:c.1138delA |  |
| 16 | tissue | chr7 | MET | frameshift deletion | exon18:c.2428delA |  |
| 16 | tissue | chr10 | RET | frameshift deletion | exon16:c.2768delT |  |
| 16 | tissue | chr10 | PTEN | frameshift deletion | exon8:c.963delA |  |
| 16 | tissue | chr11 | ATM | frameshift deletion | exon17:c.2479delA |  |
| 16 | tissue | chr13 | BRCA2 | frameshift deletion | exon11:c.5930delT |  |
| 16 | tissue | chr17 | ERBB2 | frameshift deletion | exon21:c.2578delA |  |
| 16 | tissue | chr17 | BRCA1 | synonymous SNV | exon1:c.A30G |  |
| 16 | tissue | chr19 | STK11 | frameshift deletion | exon6:c.837delC |  |
| 16 | tissue | chr22 | CYP2D6 | nonsynonymous SNV | exon1:c.C100T |  |
| 16 | tissue | chr3 | CTNNB1 | nonsynonymous SNV | exon3:c.C98G |  |
| 16 | tissue | chr4 | FGFR3 | frameshift deletion | exon9:c.1200delC |  |
| 16 | tissue | chr5 | APC | frameshift deletion | exon14:c.2486delA |  |
| 16 | tissue | chr5 | APC | frameshift insertion | exon14:c.4033dupA |  |
| 16 | tissue | chr7 | MET | frameshift deletion | exon18:c.2428delA |  |
| 16 | tissue | chr10 | PTEN | frameshift deletion | exon8:c.963delA |  |
| 16 | tissue | chr13 | BRCA2 | frameshift deletion | exon11:c.6125delA |  |
| 16 | tissue | chr17 | TP53 | frameshift deletion | exon4:c.506delC |  |
| 16 | tissue | chr17 | BRCA1 | synonymous SNV | exon1:c.A30G |  |
| 16 | tissue | chr22 | CYP2D6 | nonsynonymous SNV | exon1:c.C100T |  |
| 17 | ctc | chr1 | MTHFR | nonsynonymous SNV | exon5:c.C788T |  |
| 17 | ctc | chr4 | KIT | frameshift insertion | exon10:c.1552dupC |  |
| 17 | ctc | chr5 | APC | nonsynonymous SNV | exon14:c.A2492G |  |
| 17 | ctc | chr5 | APC | nonsynonymous SNV | exon14:c.C2539T |  |
| 17 | ctc | chr7 | EGFR | frameshift deletion | exon15:c.1735delA |  |
| 17 | ctc | chr7 | BRAF | nonsynonymous SNV | exon15:c.T1769A |  |
| 17 | ctc | chr9 | TSC1 | nonsynonymous SNV | exon14:c.A1448G |  |
| 17 | ctc | chr9 | TSC1 | nonsynonymous SNV | exon14:c.T1382C |  |
| 17 | ctc | chr10 | RET | nonsynonymous SNV | exon11:c.T1940C |  |
| 17 | ctc | chr10 | PTEN | nonsynonymous SNV | exon7:c.A785G |  |
| 17 | ctc | chr10 | PTEN | frameshift deletion | exon8:c.963delA |  |
| 17 | ctc | chr11 | HRAS | frameshift insertion | exon3:c.226dupG |  |
| 17 | ctc | chr11 | HRAS | synonymous SNV | exon2:c.T81C |  |
| 17 | ctc | chr11 | HRAS | frameshift deletion | exon2:c.29delG |  |
| 17 | ctc | chr12 | PTPN11 | nonsynonymous SNV | exon3:c.A182G |  |
| 17 | ctc | chr12 | PTPN11 | synonymous SNV | exon13:c.A1533G |  |
| 17 | ctc | chr17 | TP53 | frameshift deletion | exon3:c.137delC |  |
| 17 | ctc | chr19 | STK11 | nonsynonymous SNV | exon8:c.A1066G |  |
| 17 | tissue | chr7 | MET | frameshift deletion | exon2:c.1138delA |  |
| 17 | tissue | chr10 | RET | frameshift deletion | exon16:c.2768delT |  |
| 17 | tissue | chr10 | PTEN | frameshift deletion | exon8:c.963delA |  |
| 17 | tissue | chr11 | HRAS | synonymous SNV | exon2:c.T81C |  |
| 17 | tissue | chr11 | ATM | frameshift deletion | exon17:c.2479delA |  |
| 17 | tissue | chr13 | RB1 | frameshift deletion | exon20:c.2048delT |  |
| 17 | tissue | chr17 | BRCA1 | synonymous SNV | exon1:c.A30G |  |
| 17 | tissue | chr4 | FGFR3 | frameshift deletion | exon9:c.1200delC |  |
| 17 | tissue | chr5 | FLT4 | frameshift deletion | exon30:c.3976delG |  |
| 17 | tissue | chr7 | EGFR | synonymous SNV | exon14:c.G1560A |  |
| 17 | tissue | chr7 | MET | frameshift deletion | exon2:c.1025delT |  |
| 17 | tissue | chr7 | MET | frameshift deletion | exon18:c.2428delA |  |
| 17 | tissue | chr9 | TSC1 | frameshift deletion | exon14:c.1743delA |  |
| 17 | tissue | chr9 | TSC1 | frameshift deletion | exon14:c.1369delT |  |
| 17 | tissue | chr10 | RET | frameshift deletion | exon16:c.2768delT |  |
| 17 | tissue | chr10 | PTEN | frameshift deletion | exon1:c.37delA |  |
| 17 | tissue | chr10 | PTEN | nonsynonymous SNV | exon5:c.G395A |  |
| 17 | tissue | chr10 | PTEN | frameshift deletion | exon8:c.963delA |  |
| 17 | tissue | chr11 | ATM | frameshift deletion | exon17:c.2479delA |  |
| 17 | tissue | chr13 | RB1 | frameshift deletion | exon17:c.1581delT |  |
| 17 | tissue | chr16 | CDH1 | synonymous SNV | exon11:c.T1665C |  |
| 17 | tissue | chr17 | ERBB2 | frameshift deletion | exon21:c.2578delA |  |
| 17 | tissue | chr17 | BRCA1 | synonymous SNV | exon1:c.A30G |  |
| 18 | ctc | chr1 | MTHFR | nonsynonymous SNV | exon5:c.C788T |  |
| 18 | ctc | chr1 | MTHFR | nonsynonymous SNV | exon5:c.G748C |  |
| 18 | ctc | chr2 | IDH1 | frameshift deletion | exon4:c.347delA |  |
| 18 | ctc | chr2 | UGT1A1 | synonymous SNV | exon1:c.T294C |  |
| 18 | ctc | chr2 | UGT1A1 | frameshift deletion | exon1:c.296delT |  |
| 18 | ctc | chr4 | FGFR3 | nonsynonymous SNV | exon9:c.T1184C |  |
| 18 | ctc | chr4 | FGFR3 | frameshift deletion | exon9:c.1200delC |  |
| 18 | ctc | chr6 | TPMT | frameshift deletion | exon8:c.663delT |  |
| 18 | ctc | chr7 | EGFR | frameshift deletion | exon15:c.1735delA |  |
| 18 | ctc | chr7 | MET | frameshift deletion | exon2:c.1025delT |  |
| 18 | ctc | chr7 | MET | frameshift deletion | exon2:c.1138delA |  |
| 18 | ctc | chr7 | BRAF | nonsynonymous SNV | exon15:c.T1748C |  |
| 18 | ctc | chr9 | TSC1 | frameshift deletion | exon14:c.1369delT |  |
| 18 | ctc | chr10 | RET | frameshift deletion | exon16:c.2768delT |  |
| 18 | ctc | chr10 | PTEN | frameshift deletion | exon8:c.963delA |  |
| 18 | ctc | chr11 | HRAS | synonymous SNV | exon3:c.G273A |  |
| 18 | ctc | chr11 | HRAS | synonymous SNV | exon2:c.T81C |  |
| 18 | ctc | chr11 | ATM | nonsynonymous SNV | exon17:c.G2500A |  |
| 18 | ctc | chr16 | CDH1 | nonsynonymous SNV | exon8:c.C1040T |  |
| 18 | ctc | chr17 | ERBB2 | frameshift deletion | exon21:c.2578delA |  |
| 18 | ctc | chr19 | STK11 | synonymous SNV | exon4:c.G480A |  |
| 18 | ctc | chr19 | STK11 | frameshift deletion | exon6:c.837delC |  |
| 18 | ctc | chr19 | ERCC1 | frameshift deletion | exon3:c.326delG |  |
| 18 | tissue | chr1 | AKT3 | frameshift deletion | exon5:c.516delA |  |
| 18 | tissue | chr3 | VHL | nonsynonymous SNV | exon1:c.G332A |  |
| 18 | tissue | chr4 | KIT | frameshift deletion | exon2:c.126delA |  |
| 18 | tissue | chr5 | APC | frameshift deletion | exon7:c.818delT |  |
| 18 | tissue | chr7 | MET | frameshift deletion | exon2:c.488delC |  |
| 18 | tissue | chr7 | MET | frameshift deletion | exon2:c.1025delT |  |
| 18 | tissue | chr9 | TSC1 | frameshift deletion | exon14:c.1743delA |  |
| 18 | tissue | chr9 | TSC1 | frameshift deletion | exon14:c.1369delT |  |
| 18 | tissue | chr10 | PTEN | frameshift deletion | exon8:c.963delA |  |
| 18 | tissue | chr13 | BRCA2 | frameshift deletion | exon11:c.5578delA |  |
| 18 | tissue | chr13 | BRCA2 | frameshift deletion | exon11:c.6125delA |  |
| 18 | tissue | chr13 | RB1 | frameshift deletion | exon17:c.1581delT |  |
| 18 | tissue | chr13 | RB1 | frameshift deletion | exon20:c.2048delT |  |
| 18 | tissue | chr17 | TP53 | frameshift deletion | exon4:c.506delC |  |
| 18 | tissue | chr17 | TP53 | frameshift deletion | exon2:c.173delC |  |
| 18 | tissue | chr19 | STK11 | frameshift deletion | exon6:c.837delC |  |
| 19 | ctc | chr2 | UGT1A1 | frameshift deletion | exon1:c.296delT |  |
| 19 | ctc | chr5 | FLT4 | nonsynonymous SNV | exon11:c.G1492A |  |
| 19 | ctc | chr9 | TSC1 | frameshift deletion | exon14:c.1369delT |  |
| 19 | ctc | chr17 | ERBB2 | frameshift deletion | exon21:c.2578delA |  |
| 19 | tissue | chr4 | FGFR3 | frameshift deletion | exon9:c.1200delC |  |
| 19 | tissue | chr5 | APC | frameshift deletion | exon7:c.818delT |  |
| 19 | tissue | chr5 | APC | frameshift deletion | exon14:c.2549delA |  |
| 19 | tissue | chr5 | FLT4 | frameshift deletion | exon30:c.3976delG |  |
| 19 | tissue | chr7 | MET | frameshift deletion | exon2:c.488delC |  |
| 19 | tissue | chr7 | MET | frameshift deletion | exon2:c.1025delT |  |
| 19 | tissue | chr7 | MET | frameshift deletion | exon2:c.1138delA |  |
| 19 | tissue | chr9 | TSC1 | frameshift deletion | exon16:c.1968delT |  |
| 19 | tissue | chr9 | TSC1 | frameshift deletion | exon14:c.1369delT |  |
| 19 | tissue | chr10 | RET | frameshift deletion | exon16:c.2768delT |  |
| 19 | tissue | chr10 | PTEN | frameshift deletion | exon8:c.963delA |  |
| 19 | tissue | chr12 | KRAS | frameshift deletion | exon4:c.384delA |  |
| 19 | tissue | chr13 | BRCA2 | frameshift deletion | exon11:c.5930delT |  |
| 19 | tissue | chr13 | BRCA2 | frameshift deletion | exon11:c.6125delA |  |
| 19 | tissue | chr17 | TP53 | frameshift deletion | exon4:c.506delC |  |
| 19 | tissue | chr17 | ERBB2 | frameshift deletion | exon21:c.2578delA |  |
| 20 | ctc | chr4 | FGFR3 | frameshift deletion | exon9:c.1200delC |  |
| 20 | ctc | chr5 | PIK3R1 | synonymous SNV | exon6:c.A522G |  |
| 20 | ctc | chr5 | APC | synonymous SNV | exon14:c.T3246C |  |
| 20 | ctc | chr10 | RET | synonymous SNV | exon11:c.C2010T |  |
| 20 | ctc | chr10 | RET | frameshift deletion | exon16:c.2768delT |  |
| 20 | ctc | chr17 | TP53 | nonsynonymous SNV | exon3:c.G347C |  |
| 20 | ctc | chr17 | ERBB2 | frameshift deletion | exon21:c.2578delA |  |
| 20 | ctc | chr22 | CYP2D6 | nonsynonymous SNV | exon1:c.C100T |  |
| 20 | tissue | chr1 | NRAS | frameshift deletion | exon4:c.384delA |  |
| 20 | tissue | chr1 | NRAS | frameshift deletion | exon2:c.49delA |  |
| 20 | tissue | chr1 | AKT3 | frameshift deletion | exon5:c.516delA |  |
| 20 | tissue | chr2 | UGT1A1 | frameshift deletion | exon1:c.296delT |  |
| 20 | tissue | chr4 | FGFR3 | frameshift deletion | exon9:c.1200delC |  |
| 20 | tissue | chr5 | APC | frameshift deletion | exon7:c.818delT |  |
| 20 | tissue | chr5 | FLT4 | frameshift deletion | exon30:c.3976delG |  |
| 20 | tissue | chr7 | MET | nonsynonymous SNV | exon2:c.A1124G |  |
| 20 | tissue | chr9 | TSC1 | frameshift deletion | exon14:c.1369delT |  |
| 20 | tissue | chr10 | RET | frameshift deletion | exon16:c.2768delT |  |
| 20 | tissue | chr10 | PTEN | frameshift deletion | exon8:c.963delA |  |
| 20 | tissue | chr11 | HRAS | nonsynonymous SNV | exon2:c.C5T |  |
| 20 | tissue | chr11 | ATM | frameshift deletion | exon17:c.2479delA |  |
| 20 | tissue | chr13 | BRCA2 | frameshift deletion | exon11:c.5930delT |  |
| 20 | tissue | chr13 | RB1 | frameshift deletion | exon20:c.2048delT |  |
| 20 | tissue | chr17 | TP53 | frameshift deletion | exon4:c.506delC |  |
| 20 | tissue | chr17 | ERBB2 | frameshift deletion | exon21:c.2578delA |  |
| 20 | tissue | chr19 | ERCC1 | frameshift deletion | exon3:c.326delG |  |
| 20 | tissue | chr22 | CYP2D6 | nonsynonymous SNV | exon1:c.C100T |  |
| 21 | ctc | chr3 | VHL | synonymous SNV | exon1:c.C291T |  |
| 21 | ctc | chr4 | FGFR3 | frameshift deletion | exon9:c.1200delC |  |
| 21 | ctc | chr4 | FGFR3 | nonsynonymous SNV | exon9:c.G1231A |  |
| 21 | ctc | chr10 | RET | frameshift deletion | exon16:c.2768delT |  |
| 21 | ctc | chr11 | HRAS | nonsynonymous SNV | exon2:c.T23C |  |
| 21 | ctc | chr19 | STK11 | nonsynonymous SNV | exon8:c.G1045A |  |
| 21 | ctc | chr19 | XRCC1 | nonsynonymous SNV | exon6:c.G521T |  |
| 21 | tissue | chr4 | FGFR3 | frameshift deletion | exon9:c.1200delC |  |
| 21 | tissue | chr4 | KIT | frameshift deletion | exon2:c.126delA |  |
| 21 | tissue | chr9 | TSC1 | frameshift deletion | exon16:c.1968delT |  |
| 21 | tissue | chr9 | TSC1 | frameshift deletion | exon14:c.1743delA |  |
| 21 | tissue | chr10 | RET | frameshift deletion | exon16:c.2768delT |  |
| 21 | tissue | chr10 | PTEN | frameshift deletion | exon8:c.963delA |  |
| 21 | tissue | chr11 | ATM | frameshift deletion | exon17:c.2588delA |  |
| 21 | tissue | chr12 | KRAS | frameshift deletion | exon4:c.384delA |  |
| 21 | tissue | chr13 | BRCA2 | frameshift deletion | exon11:c.5930delT |  |
| 21 | tissue | chr13 | BRCA2 | frameshift deletion | exon11:c.5981delA |  |
| 21 | tissue | chr17 | TP53 | nonsynonymous SNV | exon3:c.G347T |  |
| 21 | tissue | chr17 | ERBB2 | frameshift deletion | exon21:c.2578delA |  |
| 21 | tissue | chr17 | BRCA1 | synonymous SNV | exon1:c.A30G |  |
| 21 | tissue | chr19 | STK11 | frameshift deletion | exon6:c.837delC |  |
| 22 | ctc | chr4 | FGFR3 | frameshift deletion | exon9:c.1200delC |  |
| 22 | ctc | chr10 | RET | nonsynonymous SNV | exon11:c.G1904T |  |
| 22 | ctc | chr10 | RET | synonymous SNV | exon11:c.C1951T |  |
| 22 | ctc | chr10 | RET | frameshift deletion | exon16:c.2768delT |  |
| 22 | tissue | chr1 | MTHFR | nonsynonymous SNV | exon5:c.C788T |  |
| 22 | tissue | chr1 | DPYD | nonsynonymous SNV | exon13:c.A1627G |  |
| 22 | tissue | chr1 | AKT3 | frameshift deletion | exon5:c.516delA |  |
| 22 | tissue | chr3 | VHL | nonsynonymous SNV | exon1:c.G332A |  |
| 22 | tissue | chr4 | FGFR3 | frameshift deletion | exon9:c.1200delC |  |
| 22 | tissue | chr10 | PTEN | frameshift deletion | exon8:c.963delA |  |
| 22 | tissue | chr11 | ATM | frameshift deletion | exon17:c.2479delA |  |
| 22 | tissue | chr13 | FLT3 | nonsynonymous SNV | exon20:c.T2474C |  |
| 22 | tissue | chr13 | BRCA2 | frameshift deletion | exon11:c.6125delA |  |
| 22 | tissue | chr17 | TP53 | nonsynonymous SNV | exon3:c.A380T |  |
| 22 | tissue | chr17 | BRCA1 | synonymous SNV | exon1:c.A30G |  |
| 22 | tissue | chr22 | CYP2D6 | synonymous SNV | exon1:c.C72T |  |
| 22 | tissue | chr4 | FGFR3 | frameshift deletion | exon9:c.1200delC |  |
| 22 | tissue | chr5 | APC | frameshift deletion | exon14:c.2486delA |  |
| 22 | tissue | chr7 | MET | frameshift deletion | exon2:c.1138delA |  |
| 22 | tissue | chr10 | RET | frameshift deletion | exon16:c.2768delT |  |
| 22 | tissue | chr10 | PTEN | frameshift deletion | exon8:c.963delA |  |
| 22 | tissue | chr11 | ATM | frameshift deletion | exon17:c.2479delA |  |
| 22 | tissue | chr13 | BRCA2 | synonymous SNV | exon11:c.A5688C |  |
| 22 | tissue | chr15 | MAP2K1 | frameshift insertion | exon2:c.222dupG |  |
| 22 | tissue | chr17 | ERBB2 | frameshift deletion | exon21:c.2578delA |  |
| 22 | tissue | chr19 | STK11 | frameshift deletion | exon6:c.837delC |  |
| 22 | tissue | chr19 | XRCC1 | nonsynonymous SNV | exon6:c.C580T |  |
| 23 | ctc | chr4 | FGFR3 | frameshift deletion | exon9:c.1200delC |  |
| 23 | ctc | chr7 | EGFR | frameshift insertion | exon14:c.1512dupC |  |
| 23 | ctc | chr7 | BRAF | frameshift insertion | exon15:c.1774dupA |  |
| 23 | tissue | chr1 | AKT3 | frameshift deletion | exon5:c.516delA |  |
| 23 | tissue | chr3 | VHL | nonsynonymous SNV | exon1:c.G332A |  |
| 23 | tissue | chr3 | CTNNB1 | nonsynonymous SNV | exon3:c.A95G |  |
| 23 | tissue | chr4 | FGFR3 | frameshift deletion | exon9:c.1200delC |  |
| 23 | tissue | chr7 | MET | frameshift deletion | exon18:c.2428delA |  |
| 23 | tissue | chr9 | TSC1 | frameshift deletion | exon14:c.1743delA |  |
| 23 | tissue | chr9 | TSC1 | nonsynonymous SNV | exon14:c.A1721G |  |
| 23 | tissue | chr9 | TSC1 | frameshift deletion | exon14:c.1369delT |  |
| 23 | tissue | chr9 | TSC1 | frameshift deletion | exon6:c.495delT |  |
| 23 | tissue | chr10 | PTEN | frameshift deletion | exon8:c.963delA |  |
| 23 | tissue | chr10 | FGFR2 | frameshift deletion | exon11:c.1640delA |  |
| 23 | tissue | chr11 | ATM | frameshift deletion | exon17:c.2479delA |  |
| 23 | tissue | chr17 | TP53 | nonsynonymous SNV | exon3:c.G347C |  |
| 23 | tissue | chr17 | ERBB2 | frameshift deletion | exon21:c.2578delA |  |
| 23 | tissue | chr17 | BRCA1 | synonymous SNV | exon1:c.A30G |  |
| 23 | tissue | chr19 | STK11 | nonsynonymous SNV | exon1:c.G154A |  |
| 23 | tissue | chr2 | UGT1A1 | frameshift deletion | exon1:c.296delT |  |
| 23 | tissue | chr3 | VHL | nonsynonymous SNV | exon1:c.G332A |  |
| 23 | tissue | chr3 | CTNNB1 | nonsynonymous SNV | exon3:c.A95G |  |
| 23 | tissue | chr4 | FGFR3 | frameshift deletion | exon9:c.1200delC |  |
| 23 | tissue | chr7 | MET | frameshift deletion | exon18:c.2428delA |  |
| 23 | tissue | chr10 | PTEN | frameshift deletion | exon8:c.963delA |  |
| 23 | tissue | chr11 | HRAS | synonymous SNV | exon2:c.T81C |  |
| 23 | tissue | chr13 | FLT3 | nonsynonymous SNV | exon14:c.A1763G |  |
| 23 | tissue | chr17 | TP53 | nonsynonymous SNV | exon3:c.G347C |  |
| 23 | tissue | chr17 | ERBB2 | frameshift insertion | exon20:c.2336dupC |  |
| 23 | tissue | chr17 | BRCA1 | frameshift deletion | exon1:c.61delA |  |
| 23 | tissue | chr17 | BRCA1 | synonymous SNV | exon1:c.A30G |  |
| 24 | ctc | chr3 | VHL | frameshift deletion | exon2:c.440delT |  |
| 24 | ctc | chr4 | FGFR3 | synonymous SNV | exon9:c.T1176C |  |
| 24 | ctc | chr4 | FGFR3 | frameshift deletion | exon9:c.1200delC |  |
| 24 | ctc | chr5 | PIK3R1 | synonymous SNV | exon6:c.T573C |  |
| 24 | ctc | chr5 | APC | nonsynonymous SNV | exon14:c.A3299G |  |
| 24 | ctc | chr7 | EGFR | nonframeshift deletion | exon14:c.1490_1492del | |
| 24 | ctc | chr7 | EGFR | frameshift insertion | exon14:c.1512dupC |  |
| 24 | ctc | chr7 | EGFR | frameshift deletion | exon15:c.1735delA |  |
| 24 | ctc | chr10 | RET | frameshift deletion | exon16:c.2768delT |  |
| 24 | ctc | chr11 | HRAS | nonsynonymous SNV | exon3:c.A214G |  |
| 24 | tissue | chr1 | NRAS | frameshift deletion | exon4:c.384delA |  |
| 24 | tissue | chr1 | AKT3 | frameshift deletion | exon5:c.516delA |  |
| 24 | tissue | chr3 | VHL | nonsynonymous SNV | exon1:c.G332A |  |
| 24 | tissue | chr5 | FLT4 | frameshift deletion | exon30:c.3976delG |  |
| 24 | tissue | chr7 | MET | frameshift deletion | exon2:c.1138delA |  |
| 24 | tissue | chr7 | MET | frameshift deletion | exon18:c.2428delA |  |
| 24 | tissue | chr10 | RET | frameshift deletion | exon16:c.2768delT |  |
| 24 | tissue | chr10 | PTEN | frameshift deletion | exon8:c.963delA |  |
| 24 | tissue | chr17 | TP53 | frameshift deletion | exon4:c.506delC |  |
| 24 | tissue | chr17 | TP53 | nonsynonymous SNV | exon4:c.G443T |  |
| 24 | tissue | chr17 | ERBB2 | frameshift deletion | exon21:c.2578delA |  |
| 24 | tissue | chr22 | CYP2D6 | nonsynonymous SNV | exon1:c.C100T |  |
| 25 | ctc | chr3 | VHL | nonsynonymous SNV | exon1:c.G245A |  |
| 25 | ctc | chr4 | FGFR3 | frameshift deletion | exon9:c.1200delC |  |
| 25 | ctc | chr10 | PTEN | frameshift insertion | exon7:c.787dupA |  |
| 25 | ctc | chr13 | BRCA2 | nonsynonymous SNV | exon11:c.A5627T |  |
| 25 | ctc | chr19 | STK11 | frameshift insertion | exon6:c.849_850insC |  |
| 25 | ctc | chr22 | CYP2D6 | nonsynonymous SNV | exon1:c.C100T |  |
| 25 | tissue | chr1 | NRAS | frameshift deletion | exon2:c.49delA |  |
| 25 | tissue | chr1 | AKT3 | frameshift deletion | exon5:c.516delA |  |
| 25 | tissue | chr3 | VHL | nonsynonymous SNV | exon1:c.G332A |  |
| 25 | tissue | chr4 | FGFR3 | frameshift deletion | exon9:c.1200delC |  |
| 25 | tissue | chr4 | KIT | frameshift deletion | exon17:c.2430delT |  |
| 25 | tissue | chr5 | APC | frameshift deletion | exon7:c.818delT |  |
| 25 | tissue | chr5 | FLT4 | frameshift deletion | exon30:c.3976delG |  |
| 25 | tissue | chr7 | MET | frameshift deletion | exon2:c.488delC |  |
| 25 | tissue | chr9 | TSC1 | frameshift deletion | exon14:c.1369delT |  |
| 25 | tissue | chr10 | RET | frameshift deletion | exon16:c.2768delT |  |
| 25 | tissue | chr10 | PTEN | frameshift deletion | exon8:c.963delA |  |
| 25 | tissue | chr16 | CDH1 | frameshift deletion | exon3:c.204delT |  |
| 25 | tissue | chr16 | CDH1 | frameshift deletion | exon6:c.743delT |  |
| 25 | tissue | chr17 | ERBB2 | frameshift deletion | exon21:c.2578delA |  |
| 25 | tissue | chr17 | ERBB2 | frameshift deletion | exon21:c.2641delG |  |
| 25 | tissue | chr17 | BRCA1 | synonymous SNV | exon1:c.A30G |  |
| 25 | tissue | chr22 | CYP2D6 | nonsynonymous SNV | exon1:c.C100T |  |
| 26 | ctc | chr1 | DPYD | stopgain | exon13:c.1692dupT |  |
| 26 | ctc | chr3 | CTNNB1 | frameshift deletion | exon3:c.179delC |  |
| 26 | ctc | chr4 | FGFR3 | frameshift deletion | exon9:c.1200delC |  |
| 26 | ctc | chr4 | FGFR3 | nonsynonymous SNV | exon9:c.A1235G |  |
| 26 | ctc | chr7 | EGFR | frameshift insertion | exon14:c.1512dupC |  |
| 26 | ctc | chr12 | PTPN11 | nonsynonymous SNV | exon13:c.A1478G |  |
| 26 | ctc | chr13 | BRCA2 | frameshift deletion | exon11:c.5646delA |  |
| 26 | ctc | chr16 | CDH1 | nonsynonymous SNV | exon6:c.A772G |  |
| 26 | ctc | chr19 | XRCC1 | frameshift insertion | exon6:c.548dupA |  |
| 26 | ctc | chr22 | CYP2D6 | nonsynonymous SNV | exon1:c.C100T |  |
| 26 | tissue | chr1 | AKT3 | frameshift deletion | exon5:c.516delA |  |
| 26 | tissue | chr3 | VHL | nonsynonymous SNV | exon1:c.G332A |  |
| 26 | tissue | chr4 | FGFR3 | frameshift deletion | exon9:c.1200delC |  |
| 26 | tissue | chr7 | MET | frameshift deletion | exon2:c.1138delA |  |
| 26 | tissue | chr7 | MET | frameshift deletion | exon18:c.2428delA |  |
| 26 | tissue | chr9 | TSC1 | frameshift deletion | exon14:c.1743delA |  |
| 26 | tissue | chr9 | TSC1 | frameshift deletion | exon14:c.1369delT |  |
| 26 | tissue | chr11 | ATM | frameshift deletion | exon17:c.2479delA |  |
| 26 | tissue | chr17 | TP53 | frameshift deletion | exon1:c.7delT |  |
| 26 | tissue | chr17 | ERBB2 | frameshift deletion | exon21:c.2578delA |  |
| 26 | tissue | chr17 | BRCA1 | synonymous SNV | exon1:c.A30G |  |
| 26 | tissue | chr19 | STK11 | frameshift deletion | exon6:c.837delC |  |
| 26 | tissue | chr19 | XRCC1 | frameshift deletion | exon6:c.565delG |  |
| 26 | tissue | chr22 | CYP2D6 | nonsynonymous SNV | exon1:c.C100T |  |
| 27 | ctc | chr1 | NRAS | frameshift deletion | exon4:c.359delT |  |
| 27 | ctc | chr3 | VHL | synonymous SNV | exon1:c.G252A |  |
| 27 | ctc | chr4 | FGFR3 | frameshift deletion | exon9:c.1200delC |  |
| 27 | ctc | chr5 | FLT4 | nonsynonymous SNV | exon11:c.A1477G |  |
| 27 | ctc | chr7 | EGFR | frameshift insertion | exon14:c.1512dupC |  |
| 27 | ctc | chr10 | PTEN | frameshift deletion | exon8:c.963delA |  |
| 27 | ctc | chr11 | HRAS | synonymous SNV | exon2:c.T81C |  |
| 27 | ctc | chr13 | BRCA2 | frameshift deletion | exon11:c.5646delA |  |
| 27 | ctc | chr17 | PIK3R5 | synonymous SNV | exon2:c.G18A |  |
| 27 | ctc | chr17 | BRCA1 | synonymous SNV | exon1:c.A30G |  |
| 27 | tissue | chr3 | VHL | nonsynonymous SNV | exon1:c.G332A |  |
| 27 | tissue | chr4 | FGFR3 | frameshift deletion | exon9:c.1200delC |  |
| 27 | tissue | chr4 | KIT | frameshift deletion | exon17:c.2430delT |  |
| 27 | tissue | chr5 | APC | frameshift deletion | exon7:c.818delT |  |
| 27 | tissue | chr5 | APC | frameshift deletion | exon14:c.2549delA |  |
| 27 | tissue | chr5 | FLT4 | frameshift deletion | exon30:c.3976delG |  |
| 27 | tissue | chr9 | TSC1 | frameshift deletion | exon14:c.1369delT |  |
| 27 | tissue | chr9 | TSC1 | frameshift deletion | exon11:c.999delA |  |
| 27 | tissue | chr10 | RET | frameshift deletion | exon16:c.2768delT |  |
| 27 | tissue | chr10 | PTEN | frameshift deletion | exon8:c.963delA |  |
| 27 | tissue | chr11 | HRAS | synonymous SNV | exon2:c.T81C |  |
| 27 | tissue | chr11 | ATM | frameshift deletion | exon17:c.2479delA |  |
| 27 | tissue | chr16 | CDH1 | frameshift deletion | exon6:c.743delT |  |
| 27 | tissue | chr17 | TP53 | frameshift deletion | exon4:c.506delC |  |
| 27 | tissue | chr17 | TP53 | nonsynonymous SNV | exon3:c.G351T |  |
| 27 | tissue | chr17 | ERBB2 | frameshift deletion | exon21:c.2578delA |  |
| 27 | tissue | chr17 | BRCA1 | synonymous SNV | exon1:c.A30G |  |
| 28 | ctc | chr4 | FGFR3 | synonymous SNV | exon9:c.C1185A |  |
| 28 | ctc | chr4 | FGFR3 | frameshift deletion | exon9:c.1200delC |  |
| 28 | ctc | chr7 | EGFR | nonsynonymous SNV | exon15:c.T1781C |  |
| 28 | ctc | chr9 | TSC1 | frameshift deletion | exon14:c.1369delT |  |
| 28 | ctc | chr10 | PTEN | frameshift deletion | exon8:c.902_933delATAGCATTTGCAGTATAGAGCGTGCAGATAAT | |
| 28 | ctc | chr14 | AKT1 | frameshift deletion | exon3:c.121_122delCG | |
| 28 | ctc | chr15 | MAP2K1 | nonsynonymous SNV | exon3:c.C405G |  |
| 28 | ctc | chr17 | BRCA1 | synonymous SNV | exon1:c.A30G |  |
| 28 | ctc | chr19 | STK11 | nonsynonymous SNV | exon8:c.A1043G |  |
| 28 | tissue | chr1 | MTHFR | nonsynonymous SNV | exon5:c.C788T |  |
| 28 | tissue | chr4 | FGFR3 | frameshift deletion | exon9:c.1200delC |  |
| 28 | tissue | chr5 | APC | frameshift deletion | exon14:c.2486delA |  |
| 28 | tissue | chr5 | FLT4 | frameshift deletion | exon30:c.3976delG |  |
| 28 | tissue | chr7 | MET | frameshift deletion | exon2:c.1138delA |  |
| 28 | tissue | chr7 | MET | frameshift deletion | exon18:c.2428delA |  |
| 28 | tissue | chr9 | TSC1 | frameshift deletion | exon16:c.1968delT |  |
| 28 | tissue | chr10 | RET | frameshift deletion | exon16:c.2768delT |  |
| 28 | tissue | chr10 | PTEN | frameshift deletion | exon8:c.963delA |  |
| 28 | tissue | chr11 | ATM | frameshift deletion | exon17:c.2588delA |  |
| 28 | tissue | chr13 | RB1 | frameshift deletion | exon20:c.2048delT |  |
| 28 | tissue | chr17 | TP53 | frameshift deletion | exon2:c.173delC |  |
| 28 | tissue | chr19 | ERCC1 | frameshift deletion | exon3:c.326delG |  |
| 29 | ctc | chr1 | DPYD | synonymous SNV | exon22:c.A2850G |  |
| 29 | ctc | chr2 | UGT1A1 | frameshift deletion | exon1:c.296delT |  |
| 29 | ctc | chr4 | FGFR3 | frameshift deletion | exon9:c.1200delC |  |
| 29 | ctc | chr7 | MET | frameshift deletion | exon18:c.2428delA |  |
| 29 | ctc | chr10 | RET | synonymous SNV | exon11:c.C1962T |  |
| 29 | ctc | chr10 | RET | synonymous SNV | exon11:c.T1989C |  |
| 29 | ctc | chr14 | AKT1 | synonymous SNV | exon3:c.G135A |  |
| 29 | ctc | chr17 | ERBB2 | frameshift insertion | exon20:c.2336dupC |  |
| 29 | tissue | chr1 | MTHFR | frameshift deletion | exon8:c.1390delG |  |
| 29 | tissue | chr1 | DPYD | nonsynonymous SNV | exon6:c.A496G |  |
| 29 | tissue | chr3 | VHL | nonsynonymous SNV | exon1:c.G332A |  |
| 29 | tissue | chr3 | CTNNB1 | nonsynonymous SNV | exon3:c.A95T |  |
| 29 | tissue | chr4 | KIT | frameshift insertion | exon2:c.94dupG |  |
| 29 | tissue | chr4 | KIT | frameshift deletion | exon2:c.126delA |  |
| 29 | tissue | chr7 | MET | frameshift deletion | exon2:c.1025delT |  |
| 29 | tissue | chr7 | MET | nonsynonymous SNV | exon2:c.A1124G |  |
| 29 | tissue | chr7 | MET | frameshift deletion | exon2:c.1138delA |  |
| 29 | tissue | chr7 | MET | frameshift deletion | exon18:c.2351delA |  |
| 29 | tissue | chr9 | TSC1 | frameshift deletion | exon14:c.1743delA |  |
| 29 | tissue | chr9 | TSC1 | frameshift deletion | exon11:c.999delA |  |
| 29 | tissue | chr10 | PTEN | frameshift deletion | exon8:c.963delA |  |
| 29 | tissue | chr12 | PTPN11 | nonsynonymous SNV | exon13:c.G1520C |  |
| 29 | tissue | chr13 | BRCA2 | frameshift deletion | exon11:c.5930delT |  |
| 29 | tissue | chr13 | RB1 | frameshift deletion | exon20:c.2048delT |  |
| 29 | tissue | chr17 | TP53 | frameshift insertion | exon4:c.484dupG |  |
| 29 | tissue | chr17 | ERBB2 | frameshift insertion | exon20:c.2336dupC |  |
| 29 | tissue | chr17 | ERBB2 | frameshift deletion | exon21:c.2578delA |  |
| 29 | tissue | chr17 | BRCA1 | synonymous SNV | exon1:c.A30G |  |
