# Supplementary material for: Correlation Between Circulating Tumor Cell DNA Genomic Alterations and Mesenchymal CTCs or CTC-Associated White Blood Cell Clusters in Hepatocellular Carcinoma
Source: Front Oncol. 2021 Jun 11;11:686365. doi: 10.3389/fonc.2021.686365 (PMC8226125; doi:10.3389/fonc.2021.686365)
Supplement: Supplementary file 3 [file Table_3.docx]

Table S3 The list of primers used to amplify 50 gene.

| #chr | start | end | no. | forward primer | reverse primer |
| --- | --- | --- | --- | --- | --- |
| chr1 | 98165055 | 98165077 | AMPL7153432996 | GAGGCAGCGAAGGATTTCTGAT | TGCTCATTGCTATTTAAAACAAGAATTCGT |
| chr1 | 97981291 | 97981315 | AMPL7156821305 | AGAGAAAGTTTTGGTGAGGGCAAA | CCCTCTTTTACACTCCTATTGATCTGG |
| chr1 | 243800837 | 243800863 | AMPL7157481363 | ATGTAAATTCATGAGCCCACAAACAC | GGTAAAGGCACTTTTGGGAAAGTTATTT |
| chr1 | 97547810 | 97547834 | AMPL7157732286 | CAACAGAAAATGCTTTCTGCCGTA | GAATTGAGCAACGTAGAGCAAGTTG |
| chr1 | 97915486 | 97915508 | AMPL7157774384 | GCAAAGCAACTGGCAGATTCTT | TTGGTGTCAAAGTGTCACTGAACTAA |
| chr1 | 115258614 | 115258638 | AMPL7153497589 | AGATGATCCGACAAGTGAGAGACA | GTGTGAAATGACTGAGTACAAACTGG |
| chr1 | 11856316 | 11856338 | AMPL7160397842 | ATGCCTTCACAAAGCGGAAGAA | CCTCTCCTGACTGTCATCCCTATT |
| chr1 | 11854397 | 11854419 | AMPL7154407877 | CACTCCAGCATCACTCACTTTG | GGGAGCTGAAGGACTACTACCT |
| chr1 | 115256396 | 115256425 | AMPL7154430727 | TCAGAGAAAATAATGCTCCTAGTACCTGT | TGGTGAAACCTGTTTGTTGGACATA |
| chr1 | 115252123 | 115252152 | AMPL7154669743 | TCACATCTCTACCAGAGTTAATCAACTGA | GGAAACAAGTGTGATTTGCCAACAA |
| chr1 | 20915112 | 20915232 | AMPL7161632566 | GGGCAAAGAGAAGTTGGAAGG | TCACTTTGTTAAATCACTTTTTCTCTCC |
| chr1 | 20915483 | 20915614 | AMPL7153662177 | CTCCTAGACCCTGCATCCTG | TTCTGGGCCATGTTGGTACC |
| chr1 | 20931377 | 20931506 | AMPL7156821285 | ACTCAACACACGCAACAGGA | CGATAGCAATTGCCCTGAAATCC |
| chr1 | 97539399 | 97539506 | AMPL7160491451 | ACCAACTTTACAGATTGGGAAGTTG | GGAAGGCCTCCCACTACTATTC |
| chr1 | 97862718 | 97862837 | AMPL7160491454 | ACCTTCCGTTTCATCCCTAT | TGTGACAGCTGCTACTACTACTAC |
| chr1 | 110253223 | 110253355 | AMPL7159095267 | AGAACACACATGGGTCAGGA | CACAGAACCTGTCCCTCAGAG |
| chr1 | 204516471 | 204516593 | AMPL7161761028 | CCACACAGATTAGTATTAATGGAGGAC | GTGTTACCTGTGGCAAGACTAAA |
| chr1 | 237048398 | 237048513 | AMPL7154407895 | ACACCAGTTTTATCATCTTTTGCTCAT | TCTGTTTCTACCACTTACCTTGAGA |
| chr2 | 234669092 | 234669117 | AMPL7156757767 | GGGACATGAAATAGTTGTCCTAGCA | ATCACACGCTGCAGGAAAGAAT |
| chr2 | 234668755 | 234668785 | AMPL7153534904 | ACCTAATAAAGCTCCACCTTCTTTATCTCT | CTTTGCTCCTGCCAGAGGTT |
| chr2 | 234665697 | 234665720 | AMPL7153659452 | GGAACAGCAAGGGATCCAGAATG | AGCTTCCTGGGCACAGAAAATT |
| chr2 | 209113054 | 209113082 | AMPL7154443611 | AAAAACATGCAAAATCACATTATTGCCA | ATCACCAAATGGCACCATACGA |
| chr2 | 38298087 | 38298213 | AMPL7158544046 | GCATCTTAGAAAGTTCTTCGCCAAT | TGTCAACCAGTGGTCTGTGAAT |
| chr2 | 234665549 | 234665669 | AMPL7158453911 | AGGATGTGTTATCTCACCAGAAC | ACTTCCTTTGATGTTCTCAAATTG |
| chr3 | 10188198 | 10188220 | AMPL7153026146 | GTCACCTTTGGCTCTTCAGAGA | CCTGTACTTACCACAACAACCTTATCTTTT |
| chr3 | 41266053 | 41266075 | AMPL7153774048 | ACAGAAAAGCGGCTGTTAGTCA | GTGAAGGACTGAGAAAATCCCTGTT |
| chr3 | 178951952 | 178951974 | AMPL7153804929 | TCAATGATGCTTGGCTCTGGAA | TGTGGAAGATCCAATCCATTTTTGTTG |
| chr3 | 178936026 | 178936053 | AMPL7154441334 | TAAGGGAAAATGACAAAGAACAGCTCA | ATGCTGAGATCAGCCAAATTCAGTTA |
| chr3 | 10183744 | 10183766 | AMPL7154484533 | CTCCCAGGTCATCTTCTGCAAT | CGTACCTCGGTAGCTGTGGAT |
| chr3 | 14187344 | 14187450 | AMPL7153659461 | AGTTTGCCTTCTCAGCAGAGA | TCCCACCTGTTCCCATTTGAG |
| chr3 | 124456699 | 124456826 | AMPL7157774387 | TGAGACAGTTGGGAGAGTGAAG | GCATAAGCCTGAGAAGCTTCG |
| chr4 | 55593342 | 55593365 | AMPL7155397205 | TCCTGCCAAAGTTTGTGATTCCA | TGGAGAGAGAACAAATAAATGGTTACCTG |
| chr4 | 55140956 | 55140978 | AMPL7153012909 | GGTGCACTGGGACTTTGGTAAT | TCCATCTCTTGGAAACTCCCATCT |
| chr4 | 1807811 | 1807831 | AMPL7153021957 | CTGGTGACCGAGGACAACGT | CAGGCGTCCTACTGGCAT |
| chr4 | 55152043 | 55152066 | AMPL7156047948 | CCTGGCACAAGGAAAAATTGTGA | GGCACCGAATCTCTAGAAGCAAC |
| chr4 | 55972919 | 55972942 | AMPL7153057982 | AGTCCTCCACACTTCTCCATTCT | TGCGTTGGAAGTTATTTCTAAGAACAGAAT |
| chr4 | 55599208 | 55599237 | AMPL7153074669 | GGTTTTCTTTTCTCCTCCAACCTAATAGT | GTCAAGCAGAGAATGGGTACTCA |
| chr4 | 55593509 | 55593534 | AMPL7153134108 | TCTCTCCAGAGTGCTCTAATGACTG | CTCCCATTTGTGATCATAAGGAAGTTG |
| chr4 | 55594152 | 55594181 | AMPL7153479414 | CTAAAATGCATGTTTCCAATTTTAGCGAG | CCTGACAGACAATAAAAGGCAGCTT |
| chr4 | 55144066 | 55144088 | AMPL7154430734 | CGGCCAGATCCAGTGAAAAACA | CTGATTGAACAGTTTTCACAACCACAT |
| chr4 | 55592101 | 55592131 | AMPL7154441335 | GAGTTCTATAGATTCTAGTGCATTCAAGCA | GACTGATATGGTAGACAGAGCCTAAAC |
| chr4 | 1808229 | 1808249 | AMPL7154483888 | GTCCTGGGAGTCTCAGGACA | CCTTCAGCAGCTTGAAGAGCT |
| chr4 | 55144474 | 55144502 | AMPL7154669759 | GGCTTTTCTGTTCTTCATTTTCATACCC | AGATATCCAGCTCTTTCTTTGGCTTC |
| chr4 | 55561663 | 55561686 | AMPL7154669760 | TGTTTTTCTTGGCAGGCTCTTCT | TCATTCGTTTCATCCAGGATCTCAAAA |
| chr4 | 55595419 | 55595443 | AMPL7154669762 | ACCACCCTTGGGTATTTTTATGGG | CCTGCTTTGAACAAATAAATGAATCACGTT |
| chr4 | 55597360 | 55597390 | AMPL7154669763 | AAACTTTACATGACTTTCCTCAAATTGGTC | CTCCAGGTTTCATGTCCATGTACTC |
| chr4 | 1806097 | 1806118 | AMPL7154699009 | GTATGCAGGCATCCTCAGCTA | ACCTGGTATCTACTTTCTGTTACCTGT |
| chr5 | 112173798 | 112173826 | AMPL7155705814 | CATCAAGAGGAAGCTTAGATAGTTCTCG | ACTTCTTCCATGACTTTGGCAATCT |
| chr5 | 112175894 | 112175920 | AMPL7153010344 | AATGAAACAGAATCAGAGCAGCCTAA | TTTTTGCTTTACGTGATGACTTTGTTG |
| chr5 | 180030270 | 180030294 | AMPL7156738437 | CCCATACTCGCTGTTGTAAAACAC | CCACCCAGCCTTCTTCTCTTACTAA |
| chr5 | 112151178 | 112151207 | AMPL7157177275 | TGCTTAATTTTTAGGGTTCAACTACACGA | CCAAGAATGTCTTAGCAAAGTAGTCATG |
| chr5 | 67590960 | 67590984 | AMPL7157481464 | TTTCTTTTGCCTGCAGGATTATGC | GTCTTTCTCAGCTGGATAAGGTCTG |
| chr5 | 112175128 | 112175156 | AMPL7153171326 | TTTGTCATCAGCTGAAGATGAAATAGGA | CAGTCTGCTGGATTTGGTTCTAGG |
| chr5 | 112175295 | 112175317 | AMPL7153172222 | GCAGACTGCAGGGTTCTAGTTT | ACAGAAGTACATCTGCTAAACATGAGTG |
| chr5 | 112174508 | 112174536 | AMPL7157780131 | ACAACTTATCCTGTTTATACTGAGAGCA | GACACAAAGACTGGCTTACATTTTGATTAA |
| chr5 | 112175453 | 112175482 | AMPL7158374816 | ACTTCTGTCAGTTCACTTGATAGTTTTGA | CATGGTTTGTCCAGGGCTATCT |
| chr5 | 112175580 | 112175602 | AMPL7158374817 | CCATGCCACCAAGCAGAAGTAA | GACCCTCTGAACTGCAGCATTTA |
| chr5 | 67589046 | 67589076 | AMPL7153851128 | CTGAAATTAGGGTTTTGGGCTGATATTAAA | TCAACCACAGAACTGAAGGTTAATGG |
| chr5 | 112175728 | 112175750 | AMPL7154699025 | CCAGGTTCTTCCAGATGCTGAT | GTTTCATTCCCATTGTCATTTTCCTGAA |
| chr5 | 180050921 | 180050939 | AMPL7154743711 | CGGCTGGCCTGTACCTTA | GCAAGACCTCATGCCACAGT |
| chr5 | 7870898 | 7871016 | AMPL7154407919 | CCCATTTTTCAGTTTCACTGTTACATG | ACCTTATCGGATTCACTAATACA |
| chr5 | 79950652 | 79950782 | AMPL7153218304 | CAGTCTACGGGAAGCCTGAAA | CCAGTCCCAGACAGAACCTACTA |
| chr5 | 79951408 | 79951535 | AMPL7160831304 | CCTTCAACGCAATAAGTACGTTTG | TGCCACCTACATTAAAACACGG |
| chr6 | 43736322 | 43736344 | AMPL7157132330 | TGCTGCATTCCCATTCTCAGTC | GCTGGTTTCTGACCTGGCTATTTC |
| chr6 | 43738226 | 43738250 | AMPL7153433006 | CCCAAATCACTGTGGATTTTGGAA | CCCAAAAGCAGGTCACTCACTT |
| chr6 | 43737443 | 43737463 | AMPL7158068881 | GTGTCTGTGTGGGTGAGTGA | CCGTTCCCTCTTTGCTAGGAATATTG |
| chr6 | 18130805 | 18130837 | AMPL7163284356 | TTTTAAAAATTCATCCATTACATTTTCAGGCT | GTTGATGCTTTTGAAGAACGACATAAAAG |
| chr6 | 110759989 | 110760116 | AMPL7159444979 | AGAGAGTAGGCCAGGACTGT | TGCCTTTTGGGTAGTGGGTTAG |
| chr6 | 152382302 | 152382422 | AMPL7153659510 | ACTCAATGCTGGATGAAATGT | TTGGCTAGCATGGTTCTACC |
| chr6 | 160113797 | 160113896 | AMPL7154407929 | GCATGATCTGCGCGTTGATG | GCTGTGCTTTCTCGTCTTCAG |
| chr7 | 116411868 | 116411898 | AMPL7156899873 | TCTTTAACAAGCTCTTTCTTTCTCTCTGTT | TTCTGGAAAAGTAGCTCGGTAGTCTA |
| chr7 | 55242359 | 55242381 | AMPL7153134652 | CATGTGGCACCATCTCACAATT | AGCAAAGCAGAAACTCACATCGA |
| chr7 | 55259387 | 55259409 | AMPL7153534426 | CACAGCAGGGTCTTCTCTGTTT | TCCTTCTGCATGGTATTCTTTCTCTTC |
| chr7 | 140453074 | 140453098 | AMPL7153667985 | CCATCCACAAAATGGATCCAGACA | CTTGCTCTGATAGGAAAATGAGATCTACTG |
| chr7 | 116339601 | 116339623 | AMPL7154338470 | CAGTCGGAGGTTCACTGCATAT | AATGCAATGGATGATCTGGGAAATAAGA |
| chr7 | 140481367 | 140481392 | AMPL7154430737 | CATACTTACCATGCCACTTTCCCTT | TTCTTTTTCTGTTTGGCTTGACTTGAC |
| chr7 | 55241579 | 55241601 | AMPL7154441336 | GGTGACCCTTGTCTCTGTGTTC | TGTGCCAGGGACCTTACCTTATA |
| chr7 | 55248948 | 55248967 | AMPL7154441337 | ATGCGAAGCCACACTGACG | TCTTTGTGTTCCCGGACATAGTC |
| chr7 | 116340134 | 116340157 | AMPL7154699028 | AATAGGAGCCAGCCTGAATGATG | GTAAAAATGCTGGAGACATCTCACATTG |
| chr7 | 116423336 | 116423365 | AMPL7154699031 | ATAATTTTTGTCCTTTCTGTAGGCTGGAT | GTGGTAAACTTTTGAGTTTGCAGACT |
| chr7 | 99270423 | 99270448 | AMPL7155255269 | CACAGCAACCTTAGGTTCTAGTTCA | CGAATGCTCTACTGTCATTTCTAACCATAA |
| chr7 | 63534430 | 63534549 | AMPL7167277706 | TGCTTTACTTAGCCTAATTGGTGTT | GGACATTTCACATGTGTTATCTACCA |
| chr7 | 80532068 | 80532191 | AMPL7161922064 | TCCAGGCTCCAGTGAAGTTT | ATCCTGCTGTTTTCACGTAG |
| chr7 | 87138633 | 87138757 | AMPL7155292262 | GGCATGTATGTTGGCCTCCTT | AAGCGACTGAATGTTCAGTG |
| chr7 | 87160513 | 87160633 | AMPL7155292263 | TGCTTTGAGGAATGGTTATAAACAC | TCTGGACAAGCACTGAAAGATAAGA |
| chr7 | 87179533 | 87179659 | AMPL7153199391 | TCTGCATCAGCTGGACTGTT | AGTGAACAGTCAGTTCCTATATCCT |
| chr7 | 87233589 | 87233703 | AMPL7157732289 | TCCTAGCTCCTCTATTTAGC | CACAAGTGAGTCATATGTCTTGCT |
| chr7 | 99366035 | 99366156 | AMPL7163301681 | AAAAGCTTCTTGGTGTTTTC | AGGATGGTAAAAAGGTGCTGA |
| chr7 | 99382087 | 99382202 | AMPL7155387200 | GGAGCCATTGGCATAAAATCTATTAAATC | GGAAGGATGTGTAGGAGTCTTCT |
| chr8 | 38285845 | 38285870 | AMPL7153041121 | ATAGGAAACAGTGTCTCACGCATAC | GCACTAGCCTTGGTGAAATCTAGG |
| chr8 | 38282151 | 38282171 | AMPL7154699033 | CTACCCAGGGCCACTGTTTT | GGTTTACAACCCATCACTGGGAAA |
| chr8 | 63938717 | 63938835 | AMPL7160831316 | AGGGTTGAAAAGAGTACTCTTAC | GCAAAGAGTTTTGATGATGGAGACTAT |
| chr8 | 69389179 | 69389296 | AMPL7158526640 | ACTTTAGTGGTCTGGGTTGTAATT | ACCTAGGAGTTGAGATTTCAAGG |
| chr9 | 135775984 | 135776006 | AMPL7156738511 | GCCGCTCCGTCTTTTAGGAAAT | GAGGTCAACGAGCTCTATTTGGAA |
| chr9 | 135779031 | 135779053 | AMPL7157049078 | TCCTCACCATGGCAGCATTATG | GGCTCTCCTCCTTCAGATGAGAT |
| chr9 | 135781291 | 135781313 | AMPL7157131037 | GGGCAGGTCTATGGGAGTAAAG | AGGAGGCTTTGACTCTCCCTTTTA |
| chr9 | 135780927 | 135780954 | AMPL7157774390 | ATGGACCATTTAACACAGAAGAGAGTG | AGGAAGACTGAGGAGCTGTTAAAGA |
| chr9 | 135797079 | 135797101 | AMPL7158562411 | GAGTGGCGAGGAAGAAAACTGA | CATTACAGTATGAAAGAAAACCTGGAGACT |
| chr9 | 135797248 | 135797274 | AMPL7158562412 | GTAATGAGAACGCAAAAAGGAGACGA | TGTTTTGCACTCCTCAATCTGTCTC |
| chr9 | 135802554 | 135802584 | AMPL7154134094 | ACATATGAAATGCCTATGATATTTCAGCCA | GGAATAAGTATCATCCATTGCCCTTTTCTT |
| chr9 | 135801006 | 135801029 | AMPL7154274321 | GTGCTTGAGAGAGCTTATGCTTC | GTGTTAACTTCATACATTCATGTGAGGACT |
| chr9 | 135786757 | 135786783 | AMPL7163409896 | CCAACCACATACTAAATCTGACCCAA | ACGTCTCGGCTGATGTTGTTAAATA |
| chr9 | 135785986 | 135786008 | AMPL7154425736 | GGGAGTGAAATGTGCACGTAGT | GTGTTCATATATGTTCTGCCCTTGTCT |
| chr9 | 86900893 | 86901014 | AMPL7156821826 | GCTGTGGGTAGTCAAACATGTTTC | TCTTGATAAAGTGATTCAGGGAAT |
| chr9 | 86909494 | 86909618 | AMPL7157775153 | TGAAGGCTGTCAGAAAATATTCTTGT | AGGAGGCACTATGTGTCAGC |
| chr10 | 89711782 | 89711802 | AMPL7153002833 | TGGCTACGACCCAGTTACCA | GGTCTATAATCCAGATGATTCTTTAACAGG |
| chr10 | 123247434 | 123247456 | AMPL7156485594 | CGGCCTTTCTTCCTGGAACATT | CAGCCAGAAATGTTTTGGTAACAGAAAA |
| chr10 | 89720766 | 89720790 | AMPL7156905878 | TAGAGCGTGCAGATAATGACAAGG | CCCACAAAATGTTTAATTTAACTGACCTTAAAA |
| chr10 | 89624140 | 89624162 | AMPL7153153366 | GCCATTTCCATCCTGCAGAAGA | ATGGATACAGGTCAAGTCTAAGTCGA |
| chr10 | 43617285 | 43617308 | AMPL7158248856 | CACTCCTCTGGTTACTGAAAGCT | ACTTTGCGTGGTGTAGATATGATCAAAA |
| chr10 | 123324913 | 123324937 | AMPL7158527413 | ACAGAGAAGAGAGAGCATAGTGCT | CCCAACAATAGGACAGTGCTTATTG |
| chr10 | 89720644 | 89720673 | AMPL7160113210 | TTTTTAGGACAAAATGTTTCACTTTTGGG | ACGCTCTATACTGCAAATGCTATCG |
| chr10 | 89717671 | 89717693 | AMPL7160389109 | ACGACGGGAAGACAAGTTCATG | TTCTCCCAATGAAAGTAAAGTACAAACCTT |
| chr10 | 89692769 | 89692796 | AMPL7163239708 | GTTGCACAATATCCTTTTGAAGACCAT | CCCGATGTAATAAATATGCACATATCATTACA |
| chr10 | 43609925 | 43609944 | AMPL7163247416 | CAGATCCACTGTGCGACGA | GCCTCCGGAAGGTCATCTCA |
| chr10 | 89724990 | 89725023 | AMPL7154409853 | TAGTTTAAGATGAGTCATATTTGTGGGTTTTCA | TGGTGTCAGAATATCTATAATGATCAGGTTCAT |
| chr10 | 123257932 | 123257956 | AMPL7154699040 | GGAAGCCCAGCCATTTCTAAAATG | GAAAGACCTTTCTGATCTGGTGTCA |
| chr10 | 123274711 | 123274733 | AMPL7154699041 | TGTTCTTCATTCGGCACAGGAT | GCTAACTCTATGGCCTGCTTATCTG |
| chr10 | 123279621 | 123279641 | AMPL7154699043 | CGACCACTGTGGAGGCATTT | GGCTTTTCTGGCATGAGGTCA |
| chr10 | 89657110 | 89657214 | AMPL7153033049 | CACGCCTGGCTAAATTGATCA | TCCCAATCGTAGGCGGTAAC |
| chr10 | 96541525 | 96541641 | AMPL7155255278 | CAACCAGAGCTTGGCATATTGT | TCACTTTCCATAAAAGCAAGGTT |
| chr10 | 96701979 | 96702102 | AMPL7154415336 | TCAGCAATGGAAAGAAATGGA | CAGTGATATGGAGTAGGGTCACC |
| chr10 | 96798460 | 96798578 | AMPL7158453933 | TGGGAACAACAGAGTTAACTC | AGTTGGAATTTACATGGCAC |
| chr10 | 96811040 | 96811123 | AMPL7157775156 | AGCAGAAGAAAGAATTAGTGAGCTTTAA | ACTCTTAGATTTGCATTTTGAGACTAT |
| chr10 | 96826958 | 96827085 | AMPL7153333188 | GGTCAATGACGCAGAGTAGAGT | TCCAGCAATGGAAAGAGATGGA |
| chr10 | 98928860 | 98928989 | AMPL7160974936 | CCCACTCCACACAAGACCTAA | TAAGCTCTGCGTTAGAGAGC |
| chr11 | 108200880 | 108200910 | AMPL7152996692 | GATTTTGTAGTTCTGTTAAAGTTCATGGCT | AATTTTCAACTGCTTTACATAAGAAGCGTT |
| chr11 | 108137868 | 108137898 | AMPL7153129981 | CTTGAACATCTTTGTTTCTCTTCCTTGAAG | CGTTTGCATCACTAACACTACTATCAGG |
| chr11 | 67352548 | 67352570 | AMPL7154416198 | AGTGACTGTGTGTTGATCAGGC | AGATGCTCACATAGTTGGTGTAGATG |
| chr11 | 533760 | 533782 | AMPL7154430742 | GTTCACCTGTACTGGTGGATGT | GCCTGTTGGACATCCTGGATAC |
| chr11 | 534200 | 534220 | AMPL7154430743 | CGCCAGGCTCACCTCTATAG | AGGAGCGATGACGGAATATAAGC |
| chr11 | 108235950 | 108235978 | AMPL7154699051 | GCAGTATTTTAAGAAGGTCCTGTTGTCA | CACTCCTTTCAGTTTCTCTTGTAGTCT |
| chr11 | 108236140 | 108236162 | AMPL7154699052 | CTCAGTGTTGGTGGACAAGTGA | AGTATGTTGGCAGGTTAAAAATAAAGGCTA |
| chr11 | 4104001 | 4104123 | AMPL7153937468 | CCTCTTTCTCTTCCCTTTCCTTGTA | CCAAAGAGTGTGTTTCTCTTCTTTTTT |
| chr11 | 4113299 | 4113427 | AMPL7161949877 | ACTTCAGTGCATGTCTTAGTTGC | TCTTCCCTGCAACTGCTAGG |
| chr11 | 4115868 | 4115998 | AMPL7153659470 | GGTAGCAGGAGAGCAGCATTT | GGTTCGAATGACGTTACTCGAC |
| chr11 | 4120770 | 4120893 | AMPL7162455461 | TGGAGGGTGAGTAGGAGTTA | GCAAAGCCTTTCTTAGTTTGGC |
| chr11 | 4139069 | 4139194 | AMPL7162455463 | GGGAGCAGGATGAGATCCAAAG | ACATCACCGTCTTTTCAGGTT |
| chr11 | 4159444 | 4159566 | AMPL7157162293 | AGGGTTTGAAGACTGGGATGT | TCATCTCTATTCTCCAAAGAGCA |
| chr11 | 4159749 | 4159865 | AMPL7161949878 | GCAGGCAAAAGGAGTAATTGATTTAAA | TGACTTATTTTGCATTCCTAAAAC |
| chr11 | 69462877 | 69462965 | AMPL7154982232 | CTGTCCTACTACCGCCTCAC | CGGCATTTCCGTGGCACTAG |
| chr11 | 98164943 | 98165060 | AMPL7167277705 | TCCACACCCAAACTTTGTTTTG | AAACACAAGAAGGCCAGATG |
| chr11 | 103418071 | 103418196 | AMPL7158453940 | GGAGTTGGAGAACCAAGCATATAC | ACTGCACAACAGATGGTAAC |
| chr12 | 25398160 | 25398183 | AMPL7153129846 | CAAAGAATGGTCCTGCACCAGTA | TAAGGCCTGCTGAAAATGACTGAA |
| chr12 | 112926810 | 112926835 | AMPL7153171366 | ATGATGTTTCCTTCGTAGGTGTTGA | GGTACCTGCTCTTCTTCAATCCTG |
| chr12 | 25378531 | 25378561 | AMPL7153790092 | ATTTCAGTGTTACTTACCTGTCTTGTCTTT | AGAGTTAAGGACTCTGAAGATGTACCTATG |
| chr12 | 112888095 | 112888115 | AMPL7163241180 | GCCTCCCTTTCCAATGGACT | GACATCTCCATTCTTCTCTTTTAATTGCC |
| chr12 | 25380238 | 25380260 | AMPL7154430744 | TCCTCATGTACTGGTCCCTCAT | TGAAGTAAAAGGTGCACTGTAATAATCCAG |
| chr12 | 1756619 | 1756747 | AMPL7157848297 | CACAGTTAAACACTCAGGAAGTGG | CCATTACCCGTGCTTTAACAGG |
| chr13 | 32903472 | 32903504 | AMPL7152996488 | AAATGTGTCATGTAATCAAATAGTAGATGTGC | GTCATATTTACTTACAGCAGTAGTATCATGAGG |
| chr13 | 28610037 | 28610065 | AMPL7153092457 | TCTTCCATTATAAGAGGCATCAATGTCC | GTGAGCTTATTTCACACGTTCTTTTCT |
| chr13 | 32914325 | 32914355 | AMPL7157481524 | ATATCACCTTGTGATGTTAGTTTGGAAACT | TTTCAGAAAACACTTGTCTTGCGTTT |
| chr13 | 49033810 | 49033835 | AMPL7153172423 | TCTTATTCCCACAGTGTATCGGCTA | GCTTGATTTTCTTACTTGGTCCAAATGC |
| chr13 | 28592583 | 28592608 | AMPL7153768626 | AGTAGGAAATAGCAGCCTCACATTG | AAATAACACTCTGGTGTCATTCTTGACA |
| chr13 | 32914553 | 32914576 | AMPL7158722422 | CATTCAGACCAGCTCACAAGAGA | CTCCCTTAACTTTGTGTAAGGAACTTTCTA |
| chr13 | 32914011 | 32914033 | AMPL7154368664 | GGCCACCTGCATTTAGGATAGC | TCCAATGCCTCGTAACAACCTG |
| chr13 | 28602189 | 28602215 | AMPL7154699054 | AGTTCACACTGTGACTGAGAAAAGAC | GGAAGCCACGAGAATATTGTGAAC |
| chr13 | 28608139 | 28608169 | AMPL7154699055 | AAATGGTGAGTACGTGCATTTTAAAGATTT | GACCGGCTCCTCAGATAATGAG |
| chr13 | 48955414 | 48955441 | AMPL7154699057 | AGATTTGTCTTTCCCATGGATTCTGAA | GCTACTTACTGAGAGCCATGCAA |
| chr13 | 48619747 | 48619857 | AMPL7154059256 | AGCTTACCCAAATAAACACCC | TGGATCATAGCCTTGTTCTTTTAAACAA |
| chr13 | 95713629 | 95713747 | AMPL7159445017 | TCACATCCTCCAAATTACTTAAGC | TGTGGATATAAACAACTCTTCAATGCC |
| chr14 | 105246427 | 105246448 | AMPL7157461370 | GCCACAGAGAAGTTGTTGAGG | GTCTGACGGGTAGAGTGTGC |
| chr14 | 64700000 | 64700115 | AMPL7157848302 | GGTCATACACTGGGACCACA | AGACTGGCTCTGAGCAAAGA |
| chr15 | 66727392 | 66727414 | AMPL7153154795 | GCTGGAGGAGCTAGAGCTTGAT | GCTTGTGGGAGACCTTGAACAC |
| chr15 | 90631729 | 90631752 | AMPL7153170387 | CTTGTACTGCAGAGACAAGAGGA | GCCCATCATCTGCAAAAACATCC |
| chr15 | 66729067 | 66729092 | AMPL7153211844 | CTTCCACCTTTCTCCAGCTAATTCA | GTGTCACATACCATGTGCTCCAT |
| chr15 | 51502728 | 51502856 | AMPL7158544090 | GCCTATCCTTCTCAAAGCACATTT | TCCTATGGGTTGTCACCAAGC |
| chr15 | 51631225 | 51631347 | AMPL7158570165 | ACACCACATTGTTCTACATAGAAGTG | AACCATGGAATAGAAGCCTC |
| chr15 | 85478623 | 85478750 | AMPL7153659521 | GGAGGACTGCCCAGTGGT | GAAGGGACTGGGACACTCAC |
| chr16 | 68844075 | 68844098 | AMPL7156732294 | AGTCACCCTCACTTGGTTCTTTC | TATACCTGGAAGAGCACCTTCCAT |
| chr16 | 68855990 | 68856018 | AMPL7158068835 | ATATTCTTCTGTGAGAGGAATCCAAAGC | GGAGCAAAGTTGCCAAAATAAAACTCA |
| chr16 | 68846007 | 68846029 | AMPL7153788799 | TCCTGACTTGGTTGTGTCGATC | GAGTTATAGAATTACCGTGGTGGGATT |
| chr16 | 68835509 | 68835538 | AMPL7154699058 | GAGAAGGAATGCTCTTGTCTTTAATCTGT | GAAACCGTAGAGGCCTTTTGACT |
| chr16 | 14041860 | 14041971 | AMPL7153420027 | CCCAGACTACGGATTCTCTGG | GGACCAGGATTATACTTCTCTGACTC |
| chr16 | 69745043 | 69745172 | AMPL7154407971 | GGCTGCTTGGAGCAAAATACA | TCCTCAGAGTGGCATTCTGC |
| chr16 | 69748854 | 69748978 | AMPL7153655818 | TGGCTGTCAGAGCATTCAGA | TGTCTTCTGTCCCACAGTTC |
| chr17 | 37881131 | 37881153 | AMPL7155953622 | GGACCTGCTGAACTGGTGTATG | GGAATGGGAAGCACCCATGTAG |
| chr17 | 7573973 | 7573995 | AMPL7156003789 | CCTTGAGTTCCAAGGCCTCATT | AAATGCATGTTGCTTTTGTACCGT |
| chr17 | 41275934 | 41275964 | AMPL7156580847 | GACATGTCTTTTCTTCCCTAGTATGTAAGG | TTTATCTGCTCTTCGCGTTGAAGA |
| chr17 | 8814664 | 8814685 | AMPL7153118931 | GCTTCCTTGAGCCCTCAAACA | ATGCAGGCGATGACCCAGGAT |
| chr17 | 7577006 | 7577028 | AMPL7153153798 | CCTGCTTGCTTACCTCGCTTAG | GCTTCTCTTTTCCTATCCTGAGTAGTG |
| chr17 | 37881303 | 37881325 | AMPL7153297387 | GGATGAGCTACCTGGAGGATGT | TCCTTGGTCCTTCACCTAACCTT |
| chr17 | 7578420 | 7578441 | AMPL7154242107 | CGTCATGTGCTGTGACTGCTT | TGCCCTGACTTTCAACTCTGTC |
| chr17 | 7578187 | 7578209 | AMPL7154427840 | TCATAGGGCACCACCACACTAT | GGCCTCTGATTCCTCACTGATTG |
| chr17 | 37880125 | 37880147 | AMPL7154483886 | CCCACGCTCTTCTCACTCATAT | GGGCTTACGTCTAAGATTTCTTTGTTG |
| chr17 | 7577497 | 7577519 | AMPL7154521476 | ACCTGGAGTCTTCCAGTGTGAT | CCTCATCTTGGGCCTGTGTTATC |
| chr17 | 41209046 | 41209069 | AMPL7154861533 | CAACTTGAGGGAGGGAGCTTTAC | TGCTCCACTTCCATTGAAGGAAG |
| chr17 | 7579403 | 7579521 | AMPL7153774088 | GGTTTTCTGGGAAGGGACAGAA | ACGATATTGAACAATGGTTCACTG |
| chr17 | 37880960 | 37881096 | AMPL7158884289 | TGTGGTCTCCCATACCCTCTC | GCGGTTTTCCCGGACATG |
| chr18 | 673364 | 673393 | AMPL7157848317 | AGAATTTCACAAGCTATTCCCTCAAATCT | CTGAGCAGATAAGTGGCAGTACA |
| chr18 | 661559 | 661681 | AMPL7157848316 | TGTCAGATCGGTGGAATACTTAAAA | TGCCCATCCTATCTTTAAGTAGGC |
| chr19 | 45923621 | 45923641 | AMPL7155752729 | TCGGGAATTACGTCGCCAAA | GGCCCTGTGGTTATCAAGG |
| chr19 | 1222984 | 1223006 | AMPL7153153026 | CTGGTTCCGGAAGAAACATCCT | GTCCTGAGTGTAGATGATGTCATCC |
| chr19 | 1220365 | 1220385 | AMPL7157261527 | CCCGCAGGTACTTCTGTCAG | GCCCAGGTCGGAGATTTTGAG |
| chr19 | 45912676 | 45912698 | AMPL7157774391 | CCGCCACTGAATTCAGAGTCTG | TGGGCACCTTCAGCTTTCTTTAG |
| chr19 | 45854840 | 45854862 | AMPL7153277856 | AAGACTCAGGAGTCACCAGGAA | TCTGTTCTCTGCAGGAGGATCA |
| chr19 | 1221247 | 1221273 | AMPL7157915526 | GGGACAACATCTACAAGTTGTTTGAG | CCCAACCCTACATTTCTGCACAA |
| chr19 | 1207044 | 1207066 | AMPL7158068836 | GCTCATCGGCAAGTACCTGATG | GCAAGCCATACTTACTTCTTCACGTT |
| chr19 | 44055615 | 44055635 | AMPL7153659453 | CTGGGACCACCTGTGTTCTC | CCGCATCGTGCGTAAGGA |
| chr19 | 41522632 | 41522757 | AMPL7156272675 | CCATCCTCCAGAACTTCTCCA | ACACTGAATGACCCTGGAATCC |
| chr19 | 44057523 | 44057653 | AMPL7157775172 | CGAGTCTAGGTCTCAACCCTAC | GGGACCTTAGAAGGTGACAGTG |
| chr21 | 37518675 | 37518801 | AMPL7156822195 | GCCCAGGACCAGTGAAGAC | CGAAGCAGACGTTTACCAGTTT |
| chr21 | 46934809 | 46934909 | AMPL7154978269 | CAGGCAAGCCTGGCACATA | CCATGGTCCTGTCTGTCCTTC |
| chr22 | 42526609 | 42526630 | AMPL7153027805 | CTCACCTGGTCGAAGCAGTAT | GCCGTGATAGTGGCCATCTTC |
| chr22 | 42523845 | 42523866 | AMPL7153433133 | CTGCACATCCGGATGTAGGAT | GTTCTGTCCCGAGTATGCTCT |
| chr22 | 42524904 | 42524925 | AMPL7154856142 | CTCACGGCTTTGTCCAAGAGA | GTGATGGGCAGAAGGGCACAAAG |
| chr22 | 42524166 | 42524189 | AMPL7155255302 | CCACTCTCACCTTCTCCATCTCT | GCAAGGTCCTACGCTTCCAAAA |
