# Supplementary figures and images for: Correlation Between Circulating Tumor Cell DNA Genomic Alterations and Mesenchymal CTCs or CTC-Associated White Blood Cell Clusters in Hepatocellular Carcinoma
Source: Front Oncol. 2021 Jun 11;11:686365. doi: 10.3389/fonc.2021.686365 (PMC8226125; doi:10.3389/fonc.2021.686365)

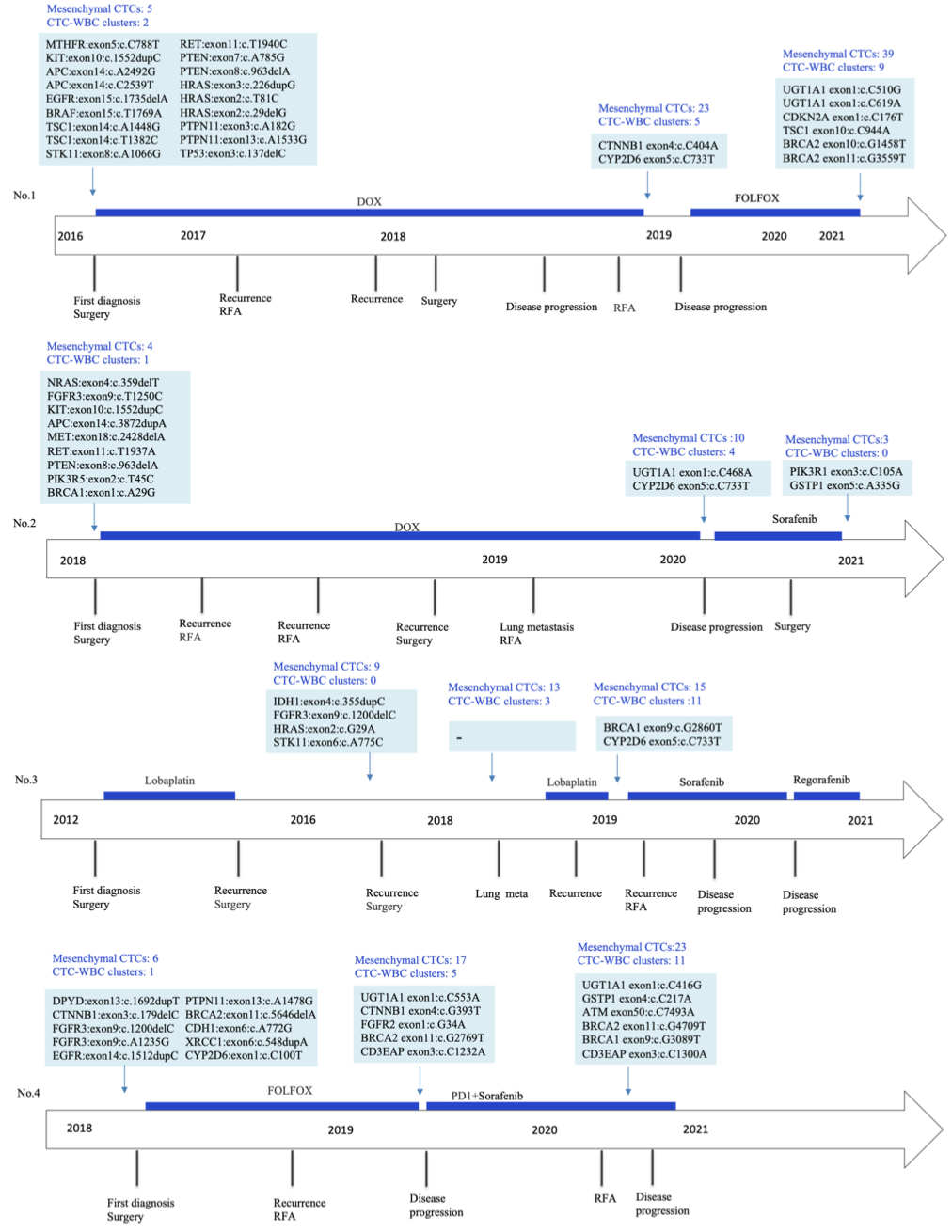

Supplement: Supplementary Figure 1 — (A, B) Case vignette monitoring the genomic alterations in circulating tumor cells and mesenchymal CTC and CTC-WBC cluster counts. [file Image_1.png]

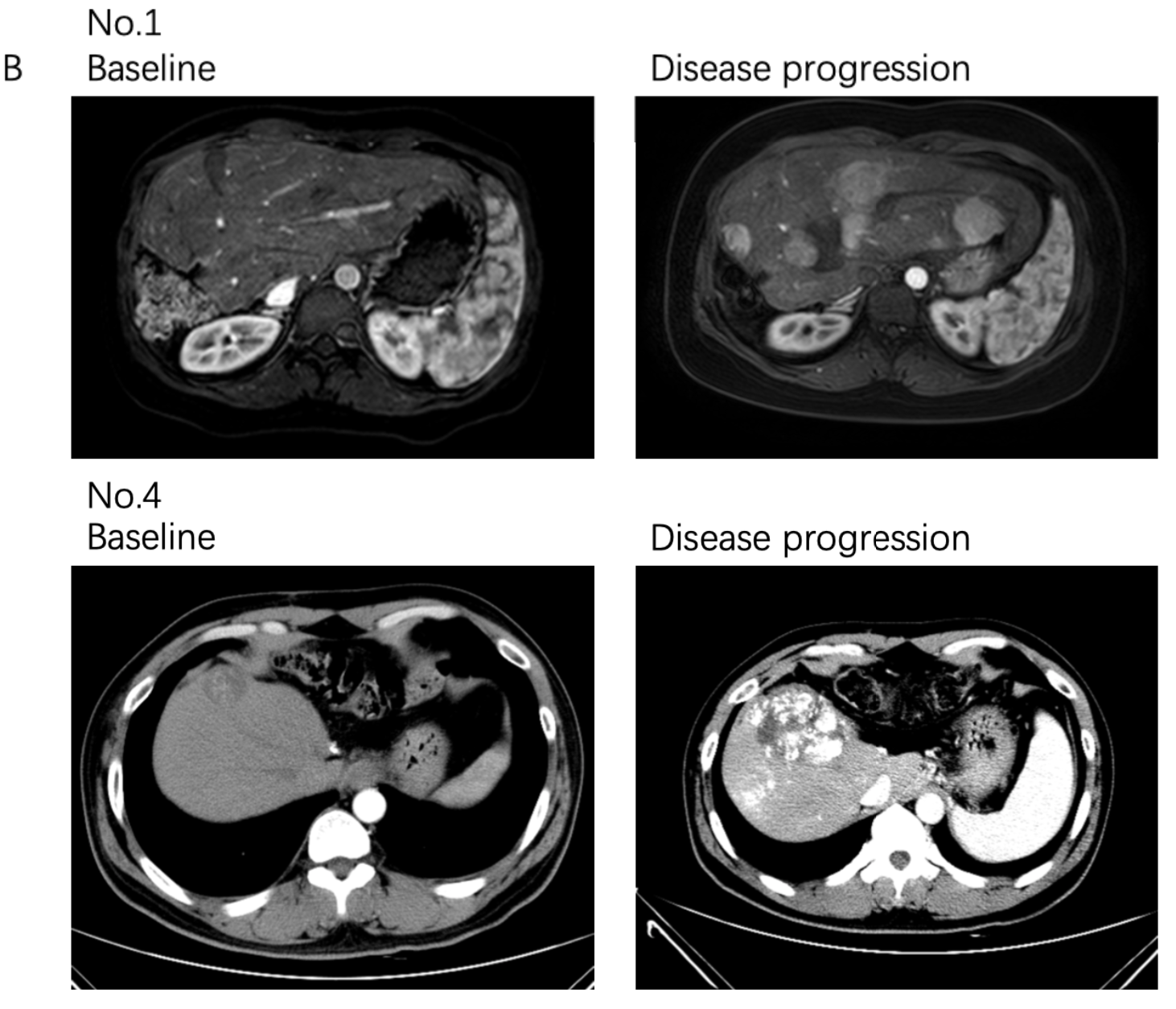

Supplement: Supplementary file 5 [file Image_2.png]
